# A robust tool for discriminative analysis and feature selection in paired samples impacts the identification of the genes essential for reprogramming lung tissue to adenocarcinoma

**DOI:** 10.1186/1471-2164-12-S3-S24

**Published:** 2011-11-30

**Authors:** Swee Heng Toh, Philip Prathipati, Efthimios Motakis, Chee Keong Kwoh, Surya Pavan Yenamandra, Vladimir A Kuznetsov

**Affiliations:** 1 Bioinformatics Institute, A-STAR, Singapore; 2 School of Computer Engineering, Nanyang Technological University, Singapore

## Abstract

**Background:**

Lung cancer is the leading cause of cancer deaths in the world. The most common type of lung cancer is lung adenocarcinoma (AC). The genetic mechanisms of the early stages and lung AC progression steps are poorly understood. There is currently no clinically applicable gene test for the early diagnosis and AC aggressiveness. Among the major reasons for the lack of reliable diagnostic biomarkers are the extraordinary heterogeneity of the cancer cells, complex and poorly understudied interactions of the AC cells with adjacent tissue and immune system, gene variation across patient cohorts, measurement variability, small sample sizes and sub-optimal analytical methods. We suggest that gene expression profiling of the primary tumours and adjacent tissues (PT-AT) handled with a rational statistical and bioinformatics strategy of biomarker prediction and validation could provide significant progress in the identification of clinical biomarkers of AC. To minimise sample-to-sample variability, repeated multivariate measurements in the same object (organ or tissue, e.g. PT-AT in lung) across patients should be designed, but prediction and validation on the genome scale with small sample size is a great methodical challenge.

**Results:**

To analyse PT-AT relationships efficiently in the statistical modelling, we propose an Extreme Class Discrimination (ECD) feature selection method that identifies a sub-set of the most discriminative variables (e.g. expressed genes). Our method consists of a paired Cross-normalization (CN) step followed by a modified sign Wilcoxon test with multivariate adjustment carried out for each variable. Using an Affymetrix U133A microarray paired dataset of 27 AC patients, we reviewed the global reprogramming of the transcriptome in human lung AC tissue versus normal lung tissue, which is associated with about 2,300 genes discriminating the tissues with 100% accuracy. Cluster analysis applied to these genes resulted in four distinct gene groups which we classified as associated with (i) up-regulated genes in the mitotic cell cycle lung AC, (ii) silenced/suppressed gene specific for normal lung tissue, (iii) cell communication and cell motility and (iv) the immune system features. The genes related to mutagenesis, specific lung cancers, early stage of AC development, tumour aggressiveness and metabolic pathway alterations and adaptations of cancer cells are strongly enriched in the AC PT-AT discriminative gene set. Two AC diagnostic biomarkers SPP1 and CENPA were successfully validated on RT-RCR tissue array. ECD method was systematically compared to several alternative methods and proved to be of better performance and as well as it was validated by comparison of the predicted gene set with literature meta-signature.

**Conclusions:**

We developed a method that identifies and selects highly discriminative variables from high dimensional data spaces of potential biomarkers based on a statistical analysis of paired samples when the number of samples is small. This method provides superior selection in comparison to conventional methods and can be widely used in different applications. Our method revealed at least 23 hundreds patho-biologically essential genes associated with the global transcriptional reprogramming of human lung epithelium cells and lung AC aggressiveness. This gene set includes many previously published AC biomarkers reflecting inherent disease complexity and specifies the mechanisms of carcinogenesis in the lung AC. SPP1, CENPA and many other PT-AT discriminative genes could be considered as the prospective diagnostic and prognostic biomarkers of lung AC.

## Background

Lung cancer is the leading cause of cancer deaths in the world [1-5]. It is very heterogeneous and high-risk mortality genetic disease. Non-Small Cell Lung Cancer (NSCLC) is the major type of human lung cancer. The most common histologic sub-type of NSCLC is lung adenocarcinoma (AC). AC is a long-term disease that takes 10 to 30 years to manifest. AC is characterized by dramatic gene expression changes in tumour cells and has a high chance of early metastasizing, due to the inherent molecular abnormalities of lung cancer cells and the presence of an extremely dense blood and lymph vessel network. Haematogenesis and lymph dissemination can spread the cancer to other lobes of the lung, liver and brain even on the early stages of the disease. Many models of AC development and progression have been discussed in the literature [1,2,6-8]. However, the genetic mechanisms of the early stages of primary lung AC progression and metastasis are poorly understood.

### Diagnosis and prognosis at early stages of the AC are a key factor for treatment outcome

Patients with lung AC have better survival rate when the localized tumour is removed at an early stage [4]. However, current clinical instrumental methods for diagnostics of lung cancers (including AC), such as CT-scan, X-ray screening are still not sufficient for reliable early diagnostics of AC and prognosis of early metastasis. In contrast to breast cancer clinical biomarkers, current status of the early diagnostic, prognostic and predictive gene expression signatures in non-small-cell lung cancer have failed to establish the usefulness of the gene markers over or above known clinical risk factors [1,2]. In particular, there is currently no clinically applicable gene test for the diagnosis and prognosis at early stage of AC [1,2,6].

The currently available AC gene signatures demonstrate substantial differences in both the number of predicted genes and the specific genes they include, mostly due to differences in the study design, assay platforms, tumour histology and patient selection protocols. Only few of the studies in the field produced detailed clinical and research protocols that were statistically and biologically validated [6-9]. Additionally, small sample size, noise and unresolved problems of massive data integration reduce the power of the test for discriminatory biological variables and clinically important predictors. Divergence and poor overlap of the AC and other lung tumours gene signatures suggests that our knowledge about nature and space dimensionality of tumour-associated genes (and potential biomarkers) is essentially incomplete [8-12]. All these questions are important in assessing the biological complexity of the tumours and the optimization of research and clinical study strategies [13]. To address them, the biological, clinical and population variations, the limited sample size and the sensitivity/specificity of the diagnostic/prognostic biomarker selection method (feature selection) should be taken into account.

### Perspective molecular markers for diagnostics, classification and staging of AC development and AC patient’s stratification

Using microarray technology, several putative gene markers of lung AC have been intensely studied and evaluated in clinical trials, for example EGFR [13] and osteopontin (SPP1) [14]. Other prospective gene markers, such as ERBB3 [15], MALAT1 [16], S100A2 [17-19], S100A6 [20], TCF21 [21-23], ABCC3 [21] and ELN3 [24] have been found and validated in independent studies. For example, S100A2 (S100 calcium binding protein A) is suspected to have a function of tumour suppressor. Therefore, it can be considered to be a promising cancer prognostic marker [19]. Currently, currently there is no clinically applied gene test for early diagnostics, prognosis and prediction of response to lung AC therapies [3].

### Paired independent and dependent design of clinical genomics study

The experimental design and the properties (or models) of the feature selection method for biomarkers prediction are two important decision steps preceding the actual data analysis. Their choice might significantly affect the sensitivity and specificity of the findings, given a high dimensionality of the data and the numerous error sources that affect them.

In contrast to studies based on unpaired data (independent patient samples for case and control), paired data experiments have not been extensively applied in feature selection of transcriptome studies aiming to biomarker identification [9,25,26]. However, due to the heterogeneous nature of the cancer genetics it is natural to consider the use of paired measurements (disease *vs*. control tissue design) originating from the same test object (a patient)[27,28]. The paired microarray data analysis compares the repeated measurements within subjects, rather than across subjects, and, in our case could increase the power of the test: multivariate paired sample design study of dependent samples obtained from the same object (a patient) could reduce the cross-population and technical variability, increase the specificity in the identification of highly discriminative features, increase the sensitivity of diagnostic and prognostic biomarker selection and facilitate disease personalization.

Haney et al. [27] showed essential improvement in the identification of differentially expressed genes when paired designs were used compared to independent sample designs. No tests were performed to determine the benefit of using different statistical procedures for paired dependent designed microarray datasets. The performance of various statistical or computational simulation methods on pair-designed microarray datasets was not sufficiently evaluated [27,28]. Interestingly, a huge improvement has been observed in DNA copy number variation (CNV) analysis when paired dependent design was used; more reliable and larger fraction of copy number variations at the genome scale were detected compared to non-paired controls [29-31]. Ideally, CNV analysis of normal tissue samples processed simultaneously with the tumour specimens would eliminate technical noise variability and batch effects [29]. Additional advantages of paired comparisons include the suppression of copy number aberrations present in both normal and tumour tissues [29]. Till recently, paired tissue samples were rarely available and acquisition of paired normal tissue did not typically fall within the purview of therapeutic surgical intervention. Consequently, normal CNV profiles are often derived from a variety of normal specimens accumulated in a laboratory or rely on data obtained from other laboratories employing the same CNV platform and/or comparison with a publicly accessible database. Heinrichs et al [30] used myeloid malignant samples to demonstrate the superiority of matched tumour and normal DNA samples (paired studies) over multiple unpaired samples with respect to reducing false discovery rates in high-resolution single nucleotide polymorphism array analysis. The advantages of paired design in CNV analysis was also suggested by Wu et al [31]. In view of the above, we propose a new statistical procedure to identify highly-discriminative genes from microarray paired dependent studies.

We suggest that analysis of gene expression data of the primary PT-AT handled with a rational computational strategy of biomarker prediction essentially facilitates personalized identification of a comprehensive set of specific AC signatures, disease-specific pathways and clinical biomarkers.

Within this framework, the primary aim of this work was to design a feature selection (e.g. class discriminative genes) method that is capable not only of identifying conventional ‘differentially expressed genes’ [32-36], but also of predicting new genes that are biologically meaningful and more clinically useful. The secondary aim was to use new methodology to identify important diagnostic and prognostic lung AC biomarkers. We achieved these aims by (i) cross-normalization of the initial dataset, (ii) selection of highly-discriminative gene sets using non-parametric tests, (iii) automatic clustering of selected genes into biologically meaningful groups, (iv) prediction of the prospective lung AC diagnostic biomarkers and (v) validation of the predictive methodology based on a comprehensive list of previously characterized gene markers of lung AC and tissue array validation of the selected discriminative genes. Further we discuss how our and other feature selection methods could be improved and used to predict novel gene markers and critical disease-associated molecular pathways.

## Results

### Cross normalization of expressed genes in paired dependent samples improves the discrimination ability of previously characterized gene markers

We introduce a function to characterise the completely discriminative variables (e.g. gene expression signals in microarray data set) which according to some additional statistical and biological criteria might be considered as the potential clinical biomarkers (see Introduction and Discussion).

Let X and Y represent the normal (adjacent) and malignant paired dependent tissue samples (X,Y), respectively. The paired signals for microarray identificator *j*,  (*i* = 1…, N; *j* = 1, …, M; X is normal class; Y is tumor class), are derived from N patients. The sub-set of *N* paired dependent signals from quantitatively distinct sample sets is called a *completely discriminative signal* (CDS) if:

or

where **1**{.} is an indicator function with value 1 if the expression in brackets holds and 0 otherwise. When , the signal is called incomplete discriminative signal (IDS).

Even if all paired samples are CDS in a given patient cohort, the increase of the number of patients (or sample size) should lead to failure of “completeness” of a given discriminative feature.

**Figure 1:**
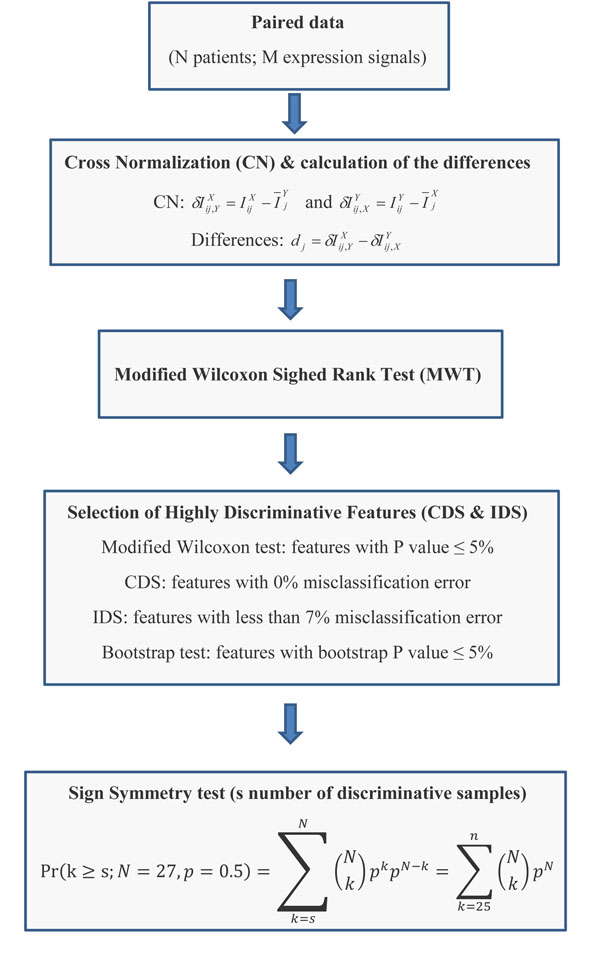
Work flow of the discriminative feature selection method

In order to select the prospective gene markers of AC and evaluate potential biomarker space of AC, we used 54 U133A Affymetrix microarray designed in pairs: lung AC versus surrounding lung tissue, which have been obtained from 27 lung AC patients [37]. The MAS5 normalized expression data and the patients’ clinical information were obtained from NCBI's GEO database (http://www.ncbi.nlm.nih.gov/geo/) using the GEO accession number GSE7670 and [37]. Figure 1 shows the work flow of our discriminative feature selection method and more details are described in Methods.

We used the sign symmetry test to estimate the p-value, *p*, of having random classification on two groups (Methods). In particular, in the extreme case of CDS (i.e. no misclassification) for our 54 samples (27 paired samples) after Bonferroni adjustment we get the p-value estimate  (i.e. no misclassification). At one (of 27 pairs) misclassification errors we get  and at two (of 27 pairs) misclassification errors we get . At the 93% confidence level, the significance of the above p-values indicates that CDS is not by chance.

In this work mostly CDS expression signals are considered as the prospective gene markers. However, a relatively weak relaxation of the extreme criteria (a few misclassifications) is also considered, but at specified statistical conditions (Methods and Discussion). In our feature selection method we used cross-normalization (CN) method followed by our modified Wilcoxon sign rank test (MWT) (Figure 1, Methods).

**Figure 2:**
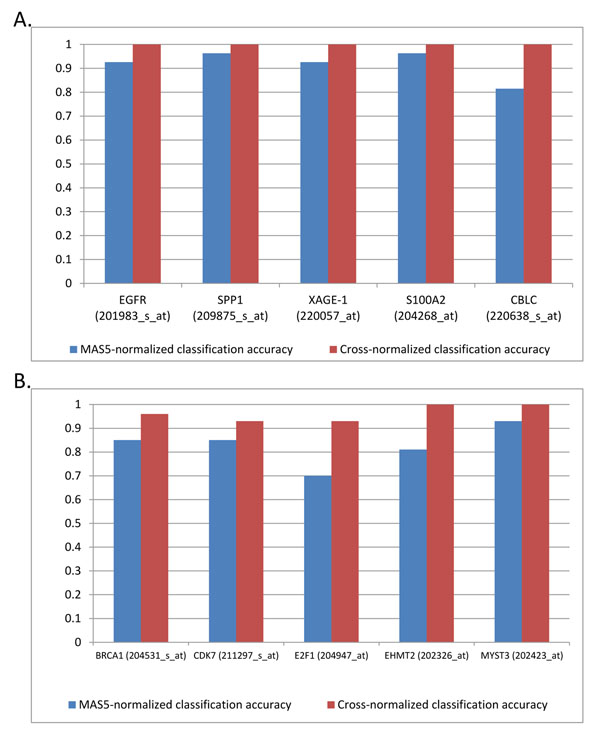
**The classification accuracy before and after cross-normalization of the original MAS5-normalized data.** Panel A depicts the improvement of the classification accuracy of selected, well-established lung AC gene markers. Panel B depicts the improvement of the classification accuracy of 5 gene signatures of lung AC [3].

To demonstrate a performance of CN and MWT with respect to its discrimination ability of the gene expression paired samples, we started with several well-defined gene markers of lung AC: EGFR [38-44], osteopontin (SPP1) [14], XAGE-1, [2,45-47], S100A2 [17-19], and CBLC [48]. The probe sets 201983_s_at (as well as 201984_s_at, 211607_x_at), 209875_s_at, 220057_at, 204268_at and 220638_s_at correspond to these genes, respectively. Figure 2 shows that the CN provides significant improvement of the separation of the paired microarray signals for all discussed gene markers of lung AC.

We also re-analysed the five-gene lung AC diagnostic signature, which was identified from protein network analysis combined with the microarray gene expression data analysis [3]. This signature includes MYST3, CDK7, BRCA1, EHMT2 and E2F1 genes, represented on the U133A chip by the probe sets 202423_at, 211297_s_at, 204531_s_at, 202326_at, 2028_s_at, respectively. We compared the results of the classification of 27 paired samples based on the initial (MAS5.0-normalized) U133A data (GSE7670) [37] and based on additional data treatment,- mutual cross-normalization (Methods).

**Figure 3:**
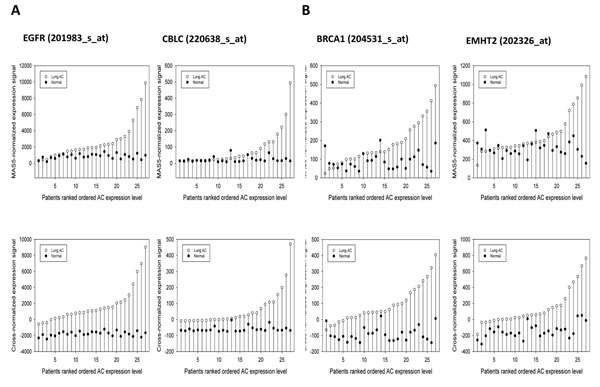
**The influence of the Cross normalization in enhancing the separability of paired samples is illustrated for selected genes from well-established Lung AC gene markers (panel A) and a 5-gene lung AC signature**[3]. The patients are rank ordered, based on the cross-normalized expression intensities of the tumor samples.

Figure 3 shows the fractions of the paired samples that correctly discriminate the AC samples *vs.* the surrounding tissue samples for the initial (MAS5.0-normalized) U133A probe sets and for that data after application of the mutual cross-normalization procedure. In some cases, the distinction between adjacent lung tissue and cancer tissue was quite strong (e.g. MYST3 (202423_at), but in general, the paired CN yielded smaller numbers of discrimination errors or even complete separation of the normal and cancer samples (e.g. 202326_at, 202423_at). Figure 3 shows four examples, which demonstrate the influence of CN in enhancing the reparability of AC vs AT in 27 sample pairs rank-ordered by the value of expression signal in AC samples.

Thus, CN provides a systematic improvement of the separation ability of the paired microarray signals for well characterized biomarkers of AC and for gene markers of the AC diagnostic signature [3] having a high discrimination ability of lung AC versus adjacent lung tissue.

### ECD identifies 2,829-signals signature and reveals the global genome reprogramming in AC cells

Our task here is to identify extremely discriminative gene expression signals (Methods, Figure 1) using 27 pairs of lung ACs and surround normal lung tissues. Using 22,283 U133A Affymetrix probe sets GSE7670 and applying consequently CN and MWT, we extracted a set of 2,967 Affymetrix U133A providing complete sample separation. The bootstrap version of ranked Welch test [35] p-value at α = 5% (Figure 1, Methods) was then applied to select 2,829 statistically significant probe sets (expression signals) (95% of the 2,967 identified initially), representing by 2,282 different GeneCards gene IDs (sheets S1a-S1c Additional file 1). This set we defined here as the PT-AT extremely discriminative classifier. These results demonstrate high sensitivity of our method and suggest a global reprogramming of lung AC cell genome in comparison with the genomes of the surrounding lung tissue cells.

### ECD signature is strongly overlapped with previously reported AC –associated gene sets and includes key functional gene subsets

Further we assessed overlap of the PT-AT ECD classifier with Significance Analysis of Microarrays (SAM) classifier derived in this work using data [49], which consists of expression profiles represented by 12,600 probe sets detected in the normal and lung tumour tissues. According to the filtering clinical and histologic data, we pre-selected 79 U95A Affymetrix microarrays profiling 17 normal lung tissue samples and 62 AC tissue samples [49]. We used SAM method [50] at 5% local false-discovery rate SAM (FDR) score, which selects 1717-probesets AC signature. 1141 of the 1717 probe sets represent 1006 up-regulated AC genes and 563 probe sets represent 495 down-regulated AC genes (sheet S1.d in Additional file 1).

**Figure 4:**
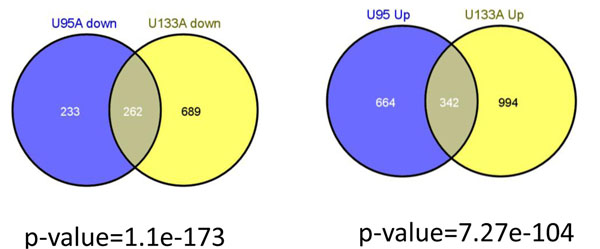
**Venn diagram analysis of the reproducibility of classifiers derived using two different Affymetrix platforms.** The high degree of overlap is interesting, given the different microarray experimental designs (the U133A classifier was derived using paired design, while the U95A classifier was derived using un-paired design). The p-value, which reflects the significance of the overlap between the 2 sets, was estimated using Fisher’s exact test [54].

These data integration results demonstrate a good agreement between SAM U95A signature and ECD U133A signature: 604 Refseq genes are common (Figure 4). 262 of the 604 genes are up-regulated and other 342 of the 604 genes are down-regulated in AC. Fold change enrichment of the common gene terms in up-regulated genes and down-regulated genes versus random co-occurrence events is 3.32 and 7.27, respectively. The hypergeometric test with extremely significant p-values (6.55e-104 and 1.71e-173 for up- and down- regulated AC genes, respectively) suggests the high agreement rate among the gene signatures. A high degree of overlapping is found in spite of differences in (i) microarray types (U133A [37] and U95A [49]), (ii) experimental designs (paired and independent sets), (iii) feature selection methods (ECD and SAM) and (iv) difference of the ethnic groups studied in [37] (Chinese) and in [49] (American).

The GO enrichment analysis, performed with the PANTHER GO tools, shows the pronounced differences between terms of up- and down-regulated GO categories of ‘Biological Process’, ‘Molecular Function’ and ‘Pathway’ annotation categories (sheet S1d in Additional file 1). The up-regulated AC gene set overrepresented by the genes involved in primary metabolic, carbohydrate metabolic process, nucleobase, nucleoside, nucleotide and nucleic acid metabolic process nucleoside, purine metabolism, de novo purine biosynthesis, cell cycle and mytosis, glycolysis, nucleic acid binding, succinate to proprionate conversion and methylmalonyl pathway. For the case of AC down-regulated categories, significant categories include immunity and defence, interleukin signalling pathway, mesoderm development, cell structure and motility, signal transduction, cell surface receptor mediated signal transduction, cell communication, intracellular signalling cascade, cell structure cell adhesion, cell motility (Figure 4, sheet S1d in Additional file 1). Importantly, one of the most strongly suppressed gene groups in lung AC is an immunity gene group. We found that these 87 ECD AC down-regulated genes could be consisting of a novel immune system signature of AC (S1e in Additional file ). We found also that 59 AC up-regulated gene subset which could be consisting of a novel cell cycle signature of AC (S1e in Additional file ). These novel signatures might be used for a future development of the diagnosis, monitoring and prognosis of the AC patients. 

Thus, our bioinformatics analysis strongly supports biological significance of at least 604 genes suggesting the clinical importance of the ECD-derived gene signature.

### Classifiers derived by PAM and EDGE methods

The application of the prediction analysis for microarrays (PAM) method [51] to the data under study produced a 77 probe set classifier, which had the higher specificity but the lower power, in comparison to other methods (see next section for further details). However, to facilitate comparison with other methods (see below), the genes were sorted with the differences between PAM up- and down- ‘Tumour scores’, and the desired numbers of genes were then selected for further studies. The optimal discovery procedure (ODP) method implemented in the EDGE program was applied to our dataset to identify the best AC-ST classifier of 996 unique, differentially expressed genes with high statistical significance (q-value<2.75e-05) (Additional file 1, Table S2.A in Additional file 2). These differential probe sets constitute about 4.4 % of the total probe sets represented on the arrays. To allow comparison with other methods, the probe sets were ranked using the ‘FDR values’ and the desired numbers of probe sets were then selected for further studies.

### Comparison of the top gene lists selected by different feature selection methods

**Figure 5:**
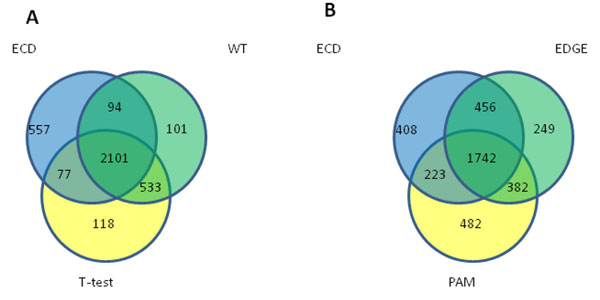
**Venn diagrams illustrating the degree of overlap between the statistically significant probe sets identified by the Modified Wilcoxon test, with the corresponding probe sets identified by other methods (WT, t-test, PAM and EDGE).** Panel A: Venn diagram illustrating the overlap between the MWT classifier and the top 2,829 differentially expressed probe sets, rank ordered using FDR values and identified using canonical approaches (t-test and WT with FDR correction). Panel B: Venn diagram depicting the overlap between the MWT classifier and the top ranked probe sets identified using computationally intensive feature selection methods.

**Figure 6:**
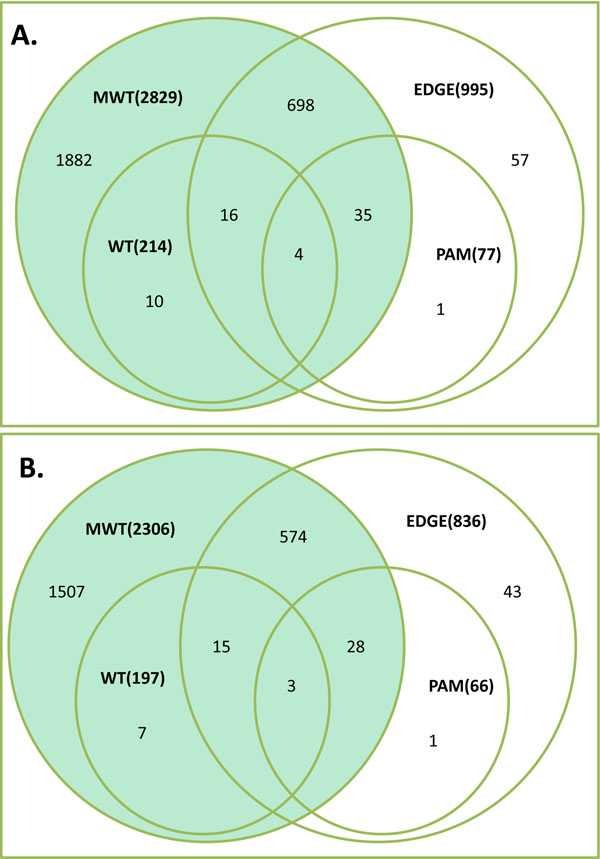
**Venn diagrams illustrating the overlap of the classifiers derived using EDGE, PAM, WT and ECD. **Panel A: Venn diagram depicting the overlap of the U133A probe sets identified as highly discriminative features. Panel B: Venn diagram depicting the overlap between the RefSeq gene symbols corresponding to the above described U133A probe sets. The numbers in parentheses are the numbers of known gene symbols present in a given subset.

To further assess the feature selection performance of ECD, we decided to overlap the top 2,829 ECD-selected probe sets with the top 2,829 probe sets derived by EDGE, PAM, Wilcoxon and t-tests. The Venn diagram in Figure 5 depicts a high fraction of overlapping (about 70% of the each group count) among the groups. However, when a complete ECD signature was compared using the best differentially expressed gene signatures of alternative methods, the ECD provides significantly larger number of the genes separating AC and AT samples (Figure 6). On the same time, each alternative method produces a proportion of the genes producing non-zero misclassification error rate (Additional file 1, Table S2.A in Additional file 2).

When the CN data were used for analysis of classification accuracy of the methods, the ECD set provides the highest, closely followed by the standard Wilcoxon sign ranked test, EDGE and the t-test. While none of 2,829 probe sets from the ECD had 2 or more misclassified sample pairs, the same-size top discriminative sets from the standard Wilcoxon sign ranked, EDGE, the t-test and PAM had 90 (3%), 111 (4%), 105(4%) and 335 (12%) probe sets with 2 or more misclassified sample pairs, respectively (Table 1). Thus, CN method improves selection accuracy for the each method used in a feature selection. The improvement effect of CN method was particularly striking for MWT, where the classification errors, in terms of the number of probe sets with two or more misclassified sample pairs, decreased from 77.4% in the initial MAS5 normalized dataset to 0% in the CN dataset.

**Table 1:** Distribution of the number of false classifications of the 27 paired samples.

	*False classifications*	*MWT*	*EDGE*	*PAM*	*Students t-test*	*Wilcoxon test*
Up-regulated in tumours	0	1628	1109	938	1087	1054
	1	0	202	211	184	187
	2	0	27	73	20	14
	3	0	1	25	0	0
	4	0	0	6	0	0
	9	0	0	1	0	0

Down- regulated in tumours	8	0	0	1	0	0
	7	0	0	1	0	0
	6	0	0	2	0	0
	5	0	0	12	0	0
	4	0	3	29	2	2
	3	0	16	67	15	10
	2	0	64	118	68	64
	1	0	317	314	343	372
	0	1201	1090	1031	1110	1126

	# of the probe sets with 2 or more misclassified pairs	0	111	335	105	90
	total # of probe sets	2829	2829	2829	2829	2829
	percent of probe sets with 2 or more misclassified pairs	0	0.04	0.12	0.04	0.03

Comparison of the classification accuracy of highly discriminative 2829 probe sets (selected using MWT on cross-normalized signal intensity values with a stringent 100% accuracy criteria and a bootstrap p-value cut-off<0.05) with top-level 2829 probe sets identified by standard Wilcoxon sign ranked test (WT), EDGE, PAM, and student&#8217s t-test. While the ECD classifier was derived using cross-normalized dataset as input, the classifiers derived using PAM, EDGE, WT and t-test used the original MAS5-normalized data as input. However the classification accuracy in terms of the number of probe sets with 2 or more anomalous fold changes was estimated using the cross-normalized dataset.

As we expected, CN also produces a substantial improvement in the classification accuracy of the top 2,829 probe sets identified using other methods. While the misclassifications, in terms of the percentage of probe sets with two or more sample pairs, in the MAS5 normalized data were 76.2, 77.3, 76.2 and 76.3 for the top 2,829 probe sets identified using by EDGE, PAM, the standard Wilcoxon test and the t-test, respectively, the false classifications for the corresponding probe sets using the CN data were 4%, 12%, 3% and 4%, respectively (Table S2.A in Additional file 2).

### The functional clusters of the lung PT-AT extremely discriminative genes

**Figure 7:**
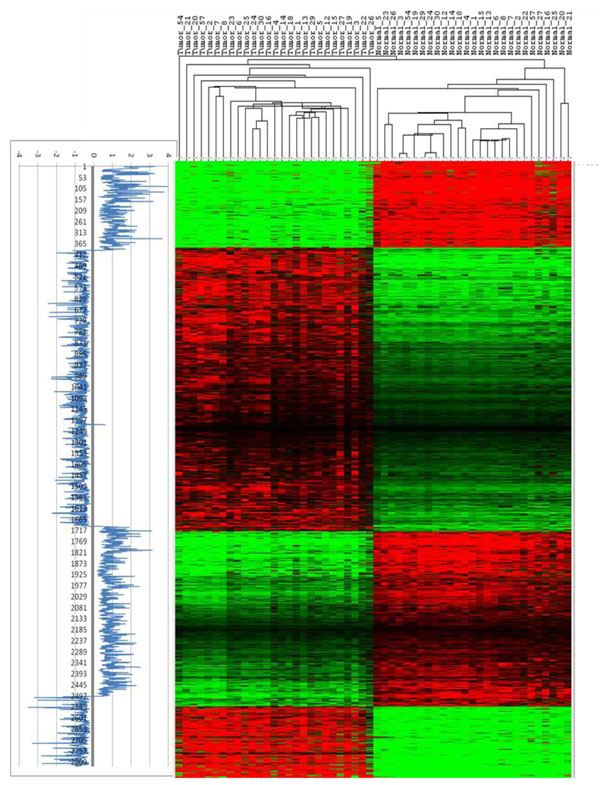
**Heatmap depicting four gene clusters which perfectly separate the AC and the surround lung tissue samples. **The similarity of the profiles was estimated using two-way hierarchical cluster analysis of the cross-normalized expression signal intensity values. The panel on the left of the heatmap shows the distribution of fold-changes of individual probe sets, ranked based on Euclidian distance metric. The distribution of the fold-change values specifies AC up- and down- regulated gene clusters.

When hierarchical clustering analysis (without sample pairing) was applied to 54 samples represented by the top-selected 2,829 probe sets, each of the feature selection method could clearly discriminate tumour samples from all normal samples (Figures S1-S4 in Additional file 3). However, CN produces fewer and more distinct gene clusters with enhanced difference between lung AC and normal samples (Figure 7; Figures S1-S4 in Additional file 3). Thus CN can be applied after the standard feature selection methods in order to increase the method performance. Furthermore, the gene clusters derived from the cross-normalized expression data are also comparatively better enriched in the top level gene ontology (GO) terms.

**Table 2:** Selected GO terms and selected genes illustrating the functional categories enriched in the clusters of discriminative genes derived using ECD (Figure 7). Several representative gene symbols are indicated in parentheses.

Cluster 1
•Inflammatory response [AGER, ALOX5, MYD88, SEPP1, SERPING1]
•Regulation of cytoskeleton organization [ABLIM1, DES, DST, NEDD9, PALLD, PRF1]
•Positive regulation of response to stimulus [C1QA, C1QB, C7, CADM1, CX3CL1, FABP4, FCER1G, MYD88, SERPING1, SLIT2]
•Wound healing [CD36, GNAQ, HBEGF, MYH10, SERPING1, THBD, VWF]
•Response to mechanical stimulus [BTG2,TIM2, CAV1,CCL2, MGP,TXNIP,TGFBR2, FOS]
•Negative regulation of cell proliferation [SFTPD, VSIG4]
•Regulation of locomotion [EGFL7,CLIC4,AGER, DLC1,ENPP2,EDN1, IL6, IL6ST, VCL]
•Complement activation [C1QA, C1QB, C7, SERPING1]
•Positive regulation of immune response [C1QA, C1QB, C7, CADM1, FCER1G, MYD88, SERPING1]
•Hormonal immune response[C1QA, C1QB, C7, CCL2, IL6, SERPING1] 1, FCER1G, MYD88, SERPING1]

**Cluster 2**

•M phase of mitotic cell cycle [AURKA, BIRC5, BUB1,CCNB1, CENPE]
•Negative regulation of intracellular transport [BARD1, GSK3B, NCBP2, NF1, TACC3]
•Microtubule-based process [CEP250, DST]
•Response to DNA damage stimulus [CSNK1D, DDB1, NONO,POLD1, TOP2A]
•Spindle organization [AURKA, BUB1B, CKS2, TTK, TUBG1, ZWINT]
•Cellular protein localization [AP1B1, ARF5, ICMT, NUP62, TIMM13, TOM1L1]
•DNA repair [APEX1, CSNK1D, DDB1, HMGB2,PARP1,POLB, POLD1,TOP2A]
•Glycosylation[ALG6,FUT2,GALNT10,MGAT4B, OGT, STT3A, UGCGL1]

**Cluster 3**

•Enzyme linked receptor protein signaling pathway [ADRB2, ARRB2,SPTBN1, TEK, TGFBR3, ZFP106]
•Metal ion homeostasis [AGTR1, C5AR1, RGN, S1PR4, TRPC6]
•Protein amino acid phosphorylation [AATK, ADRB2, TIE1, TTN, ULK2]
•Transmembrane receptor protein tyrosine kinase signaling pathway [ADRB2, CRYAB, SORBS1, TEK, ZFP106]
•Negative regulation of cell proliferation [ADAMTS8, AIF1, TENC1, TGFB1I1, TOB2]

**Cluster 4**

•Collagen fibril organization [COL3A1, COL5A2]
•Glycolysis[ALDOA, ENO1, GAPDH, PKM2, TPI1]
•Carbohydrate catabolic process [ALDOA, ENO1, FUCA1, GAPDH, TPI1]
•Extracellular structure organization [AGRN, COL11A1, COL3A1, COL5A2, ERBB2]
•Collagen metabolic process [COL3A1, MMP1, MMP11, MMP7]
•Negative regulation of cell adhesion [CDKN2A, HNRNPAB, TGFBI]
•Generation of precursor metabolites and energy [ALDOA, ENO1, GAPDH, UQCRH, UQCRQ]
•Negative regulation of protein ubiquitination [PSMA1,PSMA2, PSMB2, PSMB3, PSMB4, PSMB5]

Interestingly, GO enrichment analysis of the common biological terms reveals systematically stronger p-value of the enrichment of ECD classifier vs. alternatives. For instance, Table S2.B of Additional File 2 shows overrepresentation of cell cycle related genes containing in ECD gene signature vs. EDGE gene signature.

The hierarchical clustering analysis of the ECD signature grouped the genes into four distinct clusters: cluster 1 (397 probe sets ( p.s.); 342 Refseq genes) and cluster 3 (803 p.s.; 673 Refseq genes) contain down-regulated genes, while cluster 2 (1301 p.s.; 1101 Refseq genes) and cluster 4 ( 328 p.s. ; 264 Refseq genes ) contain the genes that are up-regulated in the lung AC vs AT (Figure 7).

Inflammatory response, regulation of cytoskeleton organization, wound healing, negative regulation of cell proliferation and regulation of immune response are some of the categories highly enriched in cluster 1, while enzyme linked receptor protein signalling pathway and metal ion homeostasis are some of the highly enriched categories in cluster 3 (Table 2). Among the highly enriched and interesting categories in clusters 2 and 4 are M-phase of mitotic cell cycle, negative regulation of intracellular transport, response to DNA damage stimulus, spindle organization, collagen fibril organization, glycolysis, focal adhesion and extracellular structure organization (Table 2). The biological categories and pathways identified here strongly suggest that the responses to external stimuli and the micro-environmental organization are highly reactive and interconnected to the progression steps of the lung AC via the mitotic cell cycle.

### The ECD-based PT-AT signatures are strongly associated with AC, other lung cancers and lung diseases

**Figure 8:**
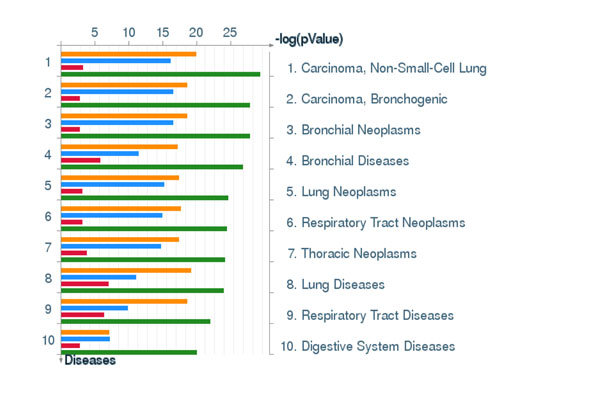
**GeneGo disease association analysis. Sorting of the GO term enrichment is done by p-values.** The p-value is estimated by GeneGo MetaCore ‘Statistically significant Diseases’ method. Yellow: cluster 1, blue: cluster 2, read: cluster 3, and green: cluster 4. Selected genes of the GO clusters are described in Table 2. Cluster 1 (top) includes genes of Inflammatory response, Regulation of cytoskeleton organization, Positive regulation of response to stimulus, Wound healing, Response to mechanical stimulus, Negative regulation of cell proliferation, Regulation of locomotion, Complement activation, Positive regulation of immune response, Hormonal immune response. These genes are suppressed in AC vs AT. Cluster 2 includes the genes of M phase of mitotic cell cycle, Negative regulation of intracellular transport, Microtubule-based process, Response to DNA damage stimulus, Spindle organization, Cellular protein localization DNA repair, Glycosylation. These genes are overexpressed in AC vs AT. Cluster 3 includes the genes of Enzyme linked receptor protein signalling pathway, Metal ion homeostasis, Protein amino acid phosphorylation, Transmembrane receptor protein tyrosine kinase signalling pathway, Negative regulation of cell proliferation. These genes are suppressed in AC vs AT. Cluster 4 includes the genes of Collagen fibril organization, Collagen metabolic process, Glycolysis, Carbohydrate catabolic process, Extracellular structure organization, Negative regulation of cell adhesion, Generation of precursor metabolites and energy, Negative regulation of protein ubiquitination. Cluster 4 includes the genes up-regulated in AC vs AT (Figure 7).

Next we studied the disease association of the four clusters of the lung AC discriminative signatures using GeneGO Diseases module, which links over 500 human diseases with genes. The GeneGO Diseases module prioritized 10 most highly enriched disease categories ranked according to p-value enrichment. The p-value enrichment values calculated with GeneGO takes into account the gene content variation between complex diseases, such as cancers and some Mendelian diseases in addition to the biased coverage of different diseases in literature. The top 10 enriched diseases for the 4 clusters of our signatures were strongly associated with lung cancers and bronchial/respiratory track diseases (Figure 8). Thus, our molecular classifier can be split into four functionally distinct groups, which are highly specific to AC and other lung tumours and lung diseases.

### The gene clusters of extremely discriminative gene signatures are associated with metabolic pathway alterations and environment adaptations

**Figure 9:**
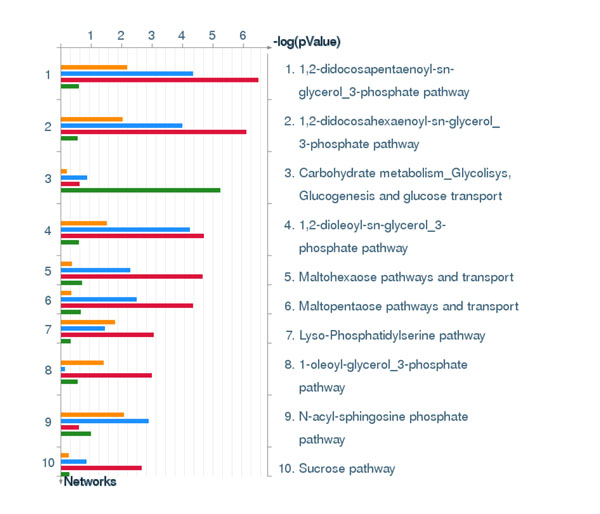
**GeneGo metabolic networks analysis.** Sorting of the GO term enrichment is done by p-values. The p-value is estimated by GeneGo MetaCore 'Statistically Significant Networks' method (see Figure 7 for details).

The metabolic pathway alterations and environment adaptations of cancer cells is an important area of anti-cancer drug discovery research. The metabolic pathways may create a phenotype that is essential for tumor cell growth and survival, altering the flux along the key metabolic pathways, such as glycolysis and glutaminolysis [52,53]. In context of the enrichment analysis of metabolic networks in Lung AC, we applied GeneGo Metabolic Networks enrichment analysis. Figure 9 shows interesting association of our clusters of discriminative genes with metabolic networks. For example, clusters 2 and 3 are enriched in glycerol-3-phosphate metabolic pathways, malto-hexaose/-pentose pathways & N-acyl-sphinngosine phosphate pathway, while cluster 4 is significantly enriched in genes of carbohydrate metabolism, glycolysis, glucogenesis and glucose transport pathway. The alterations in these metabolic pathways could lead to changes in the concentration of cognate metabolites. These metabolites could be easily detected in body fluids by non-invasive assays and hence have a great potential for the development of non-invasive diagnostic kits of lung AC.

### ECD signatures highly enriched with many previously characterized gene markers

To evaluate the signatures derived from different feature selection methods (Methods) on the 27 PT-AT paired lung AC sample U133A dataset (Dataset section in Methods), we catalogued approximately 100 references cited as either lung AC prognostic- or diagnostic- biomarkers and further manually selected 25 relevant publications and extracted set of 1,249 genes (Additional file 4). This gene set covers individual biomarkers as well as multi-gene signatures and is called here AC Meta-signature. To get a more quantitative information about expression profile of these genes in lung AC and to develop comparative analysis of this gene subset vs. other subsets (signatures), we integrated AC Meta-gene signature information presented in the Additional file 4 into our gene expression ‘master table’(sheet S1 in the Additional file 1). We used this set as a positive control to evaluate the enrichment of gene signatures studied in this work with the genes associated with lung PT-AT discrimination and the genes which could be considered as prospective clinical biomarkers.

The term enrichment of AC Meta-gene signature gene markers in various signatures was analysed using a two-sided Fisher exact test [54]. We tested enrichment of Meta-gene signature gene markers with the best gene signatures derived using EDGE, PAM and WT methods and ECD method. We found that all studied classifiers were significantly enriched with AC Meta-signature genes. However, ECD provides more complete subset found Meta-gene signature. The Fisher exact two-tail p-values sorted by significance were 4.22E-57, 1.19E-46, 5.29E-16 and 2.02E-12 for the most highly discriminative gene signatures derived using ECD, EDGE, PAM and WT, respectively.

### AC meta-signature genes and ECD PT-AT signature demonstrates high enrichment of mutagenesis sites and cancer associated proteins

Table 3 presents the counts of common fraction and the unique fractions of the genes found in the AC ECD and AC Meta-signature, respectively. All these three sub-sets are significantly enriched with the genes encoding the proteins having UniProt DB mutagenesis sites (http://www.uniprot.org/). These sites have been experimentally altered by mutagenesis. UniProt DB describes the effect of the experimental mutation of one or more amino acid(s) on the biological properties of the protein. It describes only those experiments in which a limited number of amino acid residues were altered: gross alterations in protein structure, such as the deletion of hundreds of amino acids are not described.

**Table 3:** Enriched UP_SEQ Features terms in common and unique sub-sets of PT-AT ECD-derived signature and Lung AC Meta-signature

UP_SEQ_FEATURE	Count	%	P-Value	Fold enrichment	Benjamini	FDR
**Common sub-set**						

nucleotide phosphate-binding region:ATP	59	13.3	1.60E-11	2.7	2.60E-08	2.70E-08
mutagenesis site	95	21.5	2.90E-11	2	2.40E-08	4.90E-08
binding site: ATP	36	8.1	4.20E-08	2.9	2.30E-05	7.00E-05
domain: kinesin-motor	9	2	5.20E-06	9.1	2.10E-03	8.70E-03
signal peptide	112	25.3	5.80E-06	1.5	1.90E-03	9.80E-03
domain: MCM	5	1.1	1.80E-05	27.1	4.80E-03	3.00E-02
sequence variant	317	71.7	2.30E-05	1.1	5.30E-03	3.80E-02
domain: protein kinase	27	6.1	3.50E-05	2.5	7.10E-03	5.90E-02
active site: proton acceptor	33	7.5	5.80E-05	2.2	1.00E-02	9.70E-02

**Discriminative signature: 2829 probes; unique 1862**						

mutagenesis site	271	15.4	3.70E-11	1.5	1.70E-07	6.90E-08
cross-link: Glycyl lysine isopeptide (Lys-Gly) (interchain with G-Cter in ubiquitin)	38	2.2	2.00E-05	2.1	4.40E-02	3.70E-02
binding site:substrate	50	2.8	2.90E-05	1.9	4.30E-02	5.40E-02

**Meta-signature: 1249 probes; unique 806**						

domain: protein kinase	66	8.3	4.00E-18	3.4	9.90E-15	7.00E-15
nucleotide phosphate-binding region: ATP	99	12.4	3.20E-17	2.5	4.00E-14	5.70E-14
binding site: ATP	69	8.7	1.20E-16	3.1	9.20E-14	2.00E-13
mutagenesis site	160	20.1	6.90E-16	1.9	4.20E-13	1.20E-12
active site: proton acceptor	71	8.9	2.60E-13	2.6	1.30E-10	4.60E-10
nucleotide phosphate-binding region: GTP	28	3.5	9.10E-05	2.3	3.70E-02	1.60E-01

In all, 366 UniProt proteins having mutagenesis site(s) were found with our ECD signature. Among these proteins, we found epidermal growth factor receptor (EGFR : 201983_s_at; 201984_s_at, 211607_x_at), v-erb-b2 erythroblastic leukemia viral oncogene homolog 2, neuro/glioblastoma derived oncogene homolog (avian) (ERBB2: 216836_s_at), MAD1 mitotic arrest deficient-like 1 (yeast) (MAD1L1; 204857_at), solute carrier family 22 (organic cation transporter), member 18 (SLC22A18; 204981_at) and tumour protein p73-like (TP63; 209863_s_at). Mutations in these well-known genes are associated with lung cancer, and specifically AC cells [3]. Interestingly, according to our finding all these genes are overexpressed in AC *vs.* AT (Additional file 1) and could be consists of as the prospective small signature to estimate the risk of occurrence and progression of lung AC.

Table 4 shows the statistics of the proteins having mutagenesis site(s) in the common and the unique protein subsets found in AC Meta-signature and in AC ECD. In total, the signatures include 518 proteins with the mutagenesis sites. A fraction of the proteins belonging to the common subset (21.5%) is slightly higher than that observed in the unique subsets of the Meta-signature (20.1%) and DC (15.4%). These results suggest that mutagenesis site-containing proteins can be often found in the both of our signatures. Thus, both the Meta-signature and the AC ECD signature sets are highly enriched with genes encoding the proteins having mutagenesis site(s). 98 such proteins are common for the both AC signatures and could be considered as the most prospective set of the gene markers directly related to molecular mechanisms of carcinogenesis and progression of AC.

To better assess the functional annotation of the gene markers enriched in our ECD classifiers, we will focus now on the statistical enrichment analysis of gene set with gene ontology terms (GO) [55].

**Table 4:** Statistics of ‘mutagenesis site’ term in the common and the unique protein subsets found in the AC meta-signature and AC ECD signature

Subsets	#Reported genes	# protein IDs	#Mutagenesis site	# proteins ID/# Mutagenesis site
Common genes	455	499	98	0.20
AC meta-signature only	829	813	162	0.20
ECD signature only	1852	1664	258	0.16
Total	3136	2926	518	0.19

### Not only genes of “proliferative/cell cycle signature” but many genes encoding proteins that produce signal peptides and metabolic modifications are involved in the development of AC

Interestingly, the term “signal peptide” is associated with the proteins which belong to a common fraction (112 gene symbols) (Table 3). Interestingly, this GO category term is not significantly enriched in the unique subset of our AC Meta-signature and in the unique subset of our discriminative AC signature. Reported peptides could control the essential metabolic processes and tumour development involved in local interaction of tumour with stroma, immune and endocrine system and surrounding cells of AC.

According to David Bioinformatics software, a common fraction of our discriminative AC and AC Meta-signatures is also strongly enriched with the genes encoding proteins of cell cycle, mitosis, micro-tubular based processes, DNA replication, spindle organization, regulation of cell cycle process and other GO terms which are typically represented in many “proliferative signatures” reported for many cancers. However, unique subsets are represented by more diverse sub-sets enriched with the specific GO terms. For instance, AC Meta-signature is strongly over-represented by GO terms “protein amino acid phosphorylation”, “phosphate metabolic process”, “intracellular signalling cascade”, “regulation of apoptosis”, while the unique subset of discriminative AC signature is more than 2-times enriched with the GO term “vasculature development” and 4.8 times enriched with the GO term “protein amino acid O-linked glycosylation”. The genes representing these two terms were reported in the literature to be the key players in the early stage of cancer progression, tumour dormancy and risk of tumours metastasis.

### Early diagnostic and prognostic biomarkers

EF improves significantly when the early diagnostic biomarkers reported in the literature were exclusively compared with the best discriminative genes derived using ECD and the alternative signatures. For instance, Table 5 lists 28 early diagnostic lung AC marker genes, FE of these genes measured using the Fisher’s exact two-tail p-values and the EF were 1.59e-7 and 3.76 respectively for the proposed ECD and 9.82e-7 and 6.47 respectively for EDGE. Most of EDGE selected genes are included in the ECD set (Table 5, Additional file 1). As result, ILF3, RETN1, MCM6, MUC4, supported from 2 to 4 studies, where identified by ECD method only. Interestingly, several genes of Table 5 could be considered not only prospective diagnostics biomarkers, have been reported as the prognostic biomarkers (see Table 5 references). Among such ‘dual’ biomarkers we can indicate AURKA, TOP2, ILF3, MCM6 and RFTN1 which should be test as prospective diagnostic and prognostic biomarkers.

**Table 5:** PT-AT discriminative set and its extended set are significantly enriched by gene markers reported as potential biomarkers for a diagnostic of the early stages of lung AC. The extended set was obtained by relaxing the default PT-AT ECD signature at misclassification error values to 3. More details in see Additional file 4.

Early AC diagnostic signature genes from [7]^#^	Supporting references^#^	PT-AT extreme discriminative sign. (2829 genes)	Extended set (misclassification errors to 3)	EDGE
ATP10B	[7]	YES	YES	NO
AURKA	[5][7]	YES	YES	YES
CLDN5	[7]	YES	YES	YES
COL11A1	[10][7]	YES	YES	YES
DNAI2	[7]	NO	YES	NO
FABP6	[7]	NO	NO	NO
HIGD1B	[7]	YES	YES	YES
ILF3	[5][17][2]	YES	YES	NO
IQCG	[7]	NO	NO	NO
LRRC48	[7]	NO	NO	NO
LRRC50	[7]	NO	NO	NO
MCM6	[5][7]	YES	YES	NO
MUC4	[13][7]	NO	YES	NO
RARRES2	[7][3]	YES	YES	YES
RFTN1	[22][7][4][3]	YES	YES	NO
SCG5	[7]	YES	YES	NO
SCGB1A1	[9][7]	NO	YES	YES
SFTPA2	[7]	YES	YES	NO
SFTPB	[7]	NO	YES	NO
SFTPC	[7]	YES	YES	YES
TFPI2	[7]	YES	YES	YES
TM4SF4	[7]	NO	YES	NO
TOP2A	[5][7][6]	YES	YES	YES
XAGE1A	[14][7]	YES	YES	YES
XAGE1B	[24][21][14][7]	no U133A probe sets	no U133A probe sets	no U133A probe sets
XAGE1C	[14][7]	no U133A probe sets	no U133A probe sets	no U133A probe sets
XAGE1D	[14][7]	no U133A probe sets	no U133A probe sets	no U133A probe sets
XAGE1E	[14][7]	no U133A probe sets	no U133A probe sets	no U133A probe sets

# - The references mentioned in this table correspond to the citations used in Additional file 4.

Thus, among studied feature selection methods, ECD provides the most comprehensive lists of the genes which are strongly supported by the literature as the prospective genes for biomarker discovery.

The size of the ECD-derived PT-AT discriminative signature can be extended to derive a classifier that provides better coverage of the AC Meta-signature. Many AC Meta-signature genes are well characterized as promising lung AC biomarkers (for example, BRCA1 and EHMT2, see also Figures 2 - 3). However, these gene markers are not present in ECD-derived signature. On the other hand, such gene markers could be found by WMT and the following bootstrap selection when few misclassification errors are allowed. According to previous estimates by the Sign test (Methods), we allow the feature selection method to select discriminative U133 probe sets with 0, 1 and 2 misclassification errors. In the case of two misclassification errors, ~7% (=(1-25)/27 x 100%) error rate might be acceptable according the multivariate adjusted Sing test. To reduce the risk of false positive selection, we limited the smallest fold change (ratios AC/AT or AT/AC) of mean expression signals in the paired data to higher than 1.2 and the bootstrap method p-values to less than 0.01. By applying these criteria, the ensuing PT-AT signature with 5,031 probe sets was obtained (Additional file 1). This discriminative set is also strongly enriched with known lung AC meta-signature gene markers (Fisher exact two-tail p-value=9.40E-127and FE = 2.34).

Table 5 shows that our relaxation of ECD selection criteria increase a fraction of confirmed early diagnostics genes from 62.5% (9/24) to 83.3% (20/24).

Thus, our extended PT-AT discriminative gene set (Additional set 1) and its subset of AC early diagnostic gene markers (Table 5) could be promising data sources to discover novel biomarkers and to investigate already reported promising gene markers.

### Validation of the diagnostic utility of the proposed lung AC ECD discriminative signature using tissue array assay

In order to validate the diagnostic utility of our discriminative signature we selected two genes SPP1 and CENPA, both of which showed 100% separation with strong fold change of PT-AT pairs in the U133A expression dataset. SPP1 is a well-characterized gene which is considered as a promising prognostic and diagnostic biomarker of NSCLC [13,14]. CENPA is a genomic marker for centromere activity and human diseases, including cancer [56], but it is not established as an important gene for lung AC diagnostics and prognosis.

The results of QRT-PCR tissue arrays of genes (SPP1 & CENPA) are completely concordant with the microarray analysis (Additional file 1, Figure 10); these genes perfectly discriminate 24 lung tumour samples from 24 adjacent normal lung samples. Additionally, QRT-PCR Ct values also show strong fold changes of 28.6 and 16.0 for SPP1 and CENPA, consistently with microarray expression fold-changes (Table 6), strongly suggesting a possibility to consider this gene pair as a prospective biomarker of lung AC.

**Figure10:**
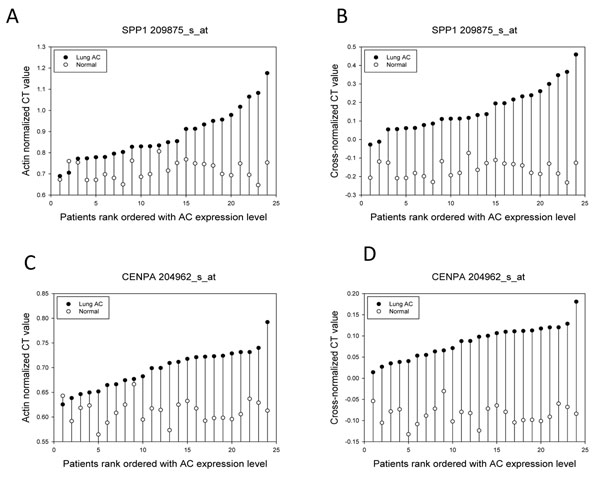
**QRT-PCR validation of potential lung AC diagnostic biomarkers identified using MWT.** The separability of the normal-Lung AC pairs of the potential lung AC gene markers identified using MWT on lung tissue QRT-PCR array is illustrated, before (A & C) and after (B & D) application of the cross-normalized expression procedure to SPP1 (A & B) and CENPA (C&D).

**Table 6:** QRT-PCR validation of potential lung AC diagnostic biomarkers identified using PT-AT ECD.

RefSeq gene (probe sets/primers)	WT		MWT		Sample pairs	Fold change (AC/N)
			
	Dis. error	P-value	Dis. error	P-value		
SPP1 (209875_s_at)	1	6.28E-06	0	1.25E-05	28	16.90
CENPA (204962_s_at)	4	9.00E-05	0	1.25E-05	28	1.41
SPP1 (primer)	1	3.03E-05	0	4.07E-05	24	28.91
CENPA (primer)	1	2.35E-05	0	4.07E-05	24	15.25

Discrimination error (Dis. Error) is the number of misclassified pairs. Fold changes for microarray was estimated as the ratio of the mean expression values of AC tissue versus adjacent normal tissue in the same tumour sample. Fold change for QRT-PCR was estimated as a ratio of the mean CT values (which were normalized using Actin-B CT values as control) of lung cancer tissues versus adjacent normal tissues in the same tumour sample. For more details of expression and QRT-PCR data normalization procedures, please refer to the materials and methods section.

## Discussion

### Advantages of the proposed ECD method

We demonstrate that the application of cross-class normalized to dependent paired data increases the distance between the objects of two classes and decreases the number of misclassification errors. The method allows us to (i) better separate the samples of the two classes (statistical issue), (ii) reduce the number of misclassification errors (pattern recognition and machine learning issues) in comparison with initial data, (iii) provide a highly sensitive and ultra-specific feature selection procedure of the discriminative features (filtering signal-noise issue) and (iv) select the most biologically reliable classifiers (discriminative genes, novel gene markers) representing the classes of interest (tumour and adjacent tissues). Our feature selection model uses consequently CN, MWT, and the bootstrap method and sign-rank tests (figure 1). It selects the statistically significant features (e.g. probe sets) focusing on the complete or almost error-less separation of the dependent paired samples when a limited number of samples is available.

### Quality control of ECD classifier

We compared the feature selection method based on ECD with the standard methods from the literature, using our aforementioned quality control approach, and then finally assess the efficiency of alternative classifiers and our method, using a comprehensive set of known gene markers of lung cancer. The efficiency of ECD classifier was successfully evaluated in several ways: (i) by the similarity in content between gene lists selected from the same data[37] (microarray U133A) using ECD and alternative feature selection methods, (ii) by the similarity in content between the gene list selected from the data [37] (microarray U133A) using ECD and the gene list selected from independent data [49] (microarray U95A) using SAM (Methods), (iii) by the enrichment of previously reported lung AC gene markers in ECD vs. AC Meta-gene signature set (iv) by the enrichment of ECD classifier with previously reported lung AC gene markers associated with early stage of the cancer progression, mutagenesis and/or risk of disease progression, (v) by RT-QPCR validation of the several predicted biomarkers.

### The similarity in content between gene lists derived from different data sets

AC signatures reported in the literature have often revealed little agreement in the gene lists produced by different methods. For example, Jeffery et al [57] found that only 8-21% of the genes were common across 10 selected features selection methods. However, the high level of agreement between ECD signature derived from data [37] and SAM signature derived from independent data [49] not only suggest a pronounced content similarity between gene lists, but also demonstrate much higher sensitivity of ECG vs SAM. Thus, our method has been shown to be highly specific and sensitive.

### Effectiveness of the classifiers derived using ECD vis-a-vis standard methods

The effectiveness of the classifiers derived using ECD and the classifiers derived using standard method was represented by the test p-values in lung AC- AT sample pairs at both individual gene and the gene group levels. Our comparative analysis of the selected gene signatures derived by different methods demonstrates the highest specificity and sensitivity of ECD. When cross-normalized data were used, the superiority of the ECD classifier at the pair level is particularly clear. All the probe sets of the ECD classifier perfectly discriminated normal lung from lung AC in all sample pairs. The selected standard methods, such as EDGE, Wilcoxon and Student’s t-test discriminated reasonably well too. Furthermore, all the methods are comparable, in terms of their ability to discriminate groups of normal and lung AC samples. However, a comparison of the classification accuracy of the various classifiers, obtained from the MAS5.0 normalized data, revealed essential improvements in the discriminative ability obtained upon the CN of the dataset. Moreover, in comparison to alternative methods, ECD classifier reveals four well-separated gene clusters. These clusters provide a clear biological interpretation of the grouped gene sets.

### Discriminative signature of lung AC is highly enriched in previously reported prospective biomarkers for early tumour diagnostics, progression and patient survival

The GO analysis suggested the enrichment of our set of predictors with oncogenes and tumour suppressor gene markers. The identification of many well-defined cancer gene markers (representing oncogenes), such as EGFR [13], osteopontin (SPP1) [13,14], ERBB3 [15], MALAT1 [16], S100A2 [17-19], S100A6 [20], ABCC3 [21] and ELN3 [24], as highly discriminative genes by the ECD is quite encouraging (sheet S1.A in Additional file 1). This identification suggests greatly enhances the clinical relevance and potential of ECD signature, as the source novel gene molecular classifiers of the AC and their biomarkers. Some selected genes with high discriminative ability, belonging to three cancer-relevant pathways, such as ATP-binding cassette (ABC) family of genes (ABCA4, ABCA8 [58]) were identified as prospective gene markers of lung AC [21] other one (ABCB1, ABCC3 and ABCF2) should be also under serious consideration. Our microarray analysis results (sheet S1.a in Additional file 1) and preliminary lung tissue array study demonstrated that the ABC gene signature, consisting of ABCA4 and ABCA8 together with ABCC3, can discriminate lung AC vs AT and be considering as a novel lung cancer diagnostic biomarker (not published).

We also identified a large number of cell adhesion and cell communication pathway genes, such as CDH3, SPP1, SPINK and CEACAM5 (sheet S1.A in Additional file 1) that are highly up-regulated in all lung AC tumours, and hence could be associated with the early metastasis of lung AC. Several tumour suppressor genes are expressed at relatively low levels across all paired samples studied. For instance, the transcription of the TCF21 gene is strongly suppressed in AC vs. adjacent lung tissue (sheet S-a in Additional file 1). TCF21 has recently been reported as being essential for the differentiation of epithelial cells adjacent to mesenchyme [22,23]. TCF21 is considered to be a candidate tumour suppressor at 6q23-q24 that is epigenetically inactivated in lung, head and neck cancers [22,23]. TCF21 was also mentioned as a promising cancer gene marker in a report that focused on the differential methylation of a short CpG-rich sequence [23]. In addition, we found essential differences in the gene expression profiles of key players in the focal adhesion pathway, such as FLNB (sheet S1.A in Additional File 1).

### Discriminative signature and meta-signatures contain genes essential for oncogenesis and tumour progression

We demonstrated that the gene sets of ECD and Meta-signature are significantly overlapped and both are highly enriched with the terms of biological processes and molecular functions which according to the literature are essential in the lung cancer and other cancers. Unique features of AC Meta-signature show a strong association with phosphate metabolic process, intracellular signalling cascade, regulation of proliferation and apoptosis. Such functions in combination with activity of the cancer-associated cell proliferative genes are often considered as the aggressiveness score of a primary tumour. The genes and their products of this unique subset might be used as a source of prognostic and predictive biomarkers.

In light of our finding of the differences between common subset and unique AD discriminative signature, we suggest that in addition to the common set of the core proliferative genes, our ECD signature includes a subset of genes encoding proteins that often undergo mutagenesis, provide a regulation of proliferation and the transition steps in the AC development. These genes and their products might be considered as a source of early and differential diagnostic biomarkers as well as the prognostic biomarkers.

### Feature selection paired design allows estimating the gene marker space discriminating PT-AT and predicts high plasticity of the transcritome in AC

Using 54 U133A microarray profiles of 27 paired samples of lung adenocarcinoma (AC) and adjacent lung tissue, we identified the global reprogramming of the transcriptional profile in human lung AC compared to normal lung tissue. Through the mutual CN of expression data belonging to dependent paired samples and using a separability score, calculated by the modified Wilcoxon signed-rank test, the method provides an essential improvement of the sensitivity of the feature selection at the highest specificity (100%) in a comparison to the alternative methods. Our method reveals expression shift of at least 2,428 genes discriminating lung AC from adjacent normal lung tissues with ~100% accuracy. Additionally, more than 2000 genes could discriminate the lung AC tumours from the normal lung tissues with ~93% accuracy.

Systematic structural and functional study of the protein peptides found in the common subset of the Meta-signature and ECD signature (as well as SAM U95 signature and ECD signature) should be useful in our understanding of the basic interactions of the tumour with stroma, surrounding tissue, immune and endocrine systems. Specifically, we suggest that immunity and signal peptides gene signatures (and their sub-networks) could be used for early diagnostics of AC and for quantitative monitoring of the adjuvant therapy. Thus, we could suggest that these genes may represent promising targets for novel specific therapies.

Non-small cell lung cancer (NSCLC) can be classified into the major subtypes, AC, and squamous cell carcinoma (SCC). A global gene expression profiling of 58 human NSCLC specimens revealed large differences between AC and SCC subtypes, with more than 1700 genes found to be differentially expressed [59]. The assignment of these genes to biological processes pointed to the deregulation of distinct sets of cell adhesion molecules. In combination with our findings this result reveals an extremely high dimensionality of the potential biomarker space and plasticity of the transcriptome in AC and SCC subtypes.

### Further method improvement and validation studies

ECD provides superior selection in comparison to conventional methods and can be widely used in different applications. However, the selection and classification performance and error estimation strongly depend on the data set size and dimensionality of the co-variate space. It is a challenging task to develop an exact model of microarray data taking into account paired dependent design and to compare the benefit of the model versus non-paired design models. Several ways to treat paired dependent data more efficiently in a parametric modelling framework have been proposed [60]. Using univariate simulation models, the authors showed essential misclassification of variance reduction in paired dependent design model, even when the sample size is relatively small. In the case of small sample size, it is important to develop simulation models based on non-parametric test (Methods, Figure 1) and to study (i) a dependence of misclassification error from the sample size, correlation patterns between paired features, (iii) a role of dimensionality of the co-variate space and other parameters of microarray data and clinical factors.

The thresholds of our feature selection procedure could be fine-tuned and corrected (e.g. by relaxation of the statistical criteria controlling FDR and modification of the fold-change cut-off value). Despite the statistically reasonable cut-off of p-value and the 100%-correct prediction criteria, we found that a large proportion of the lung cancer gene markers reported in the literature were still filtered out (Methods). Some potentially important gene markers, such as MYC and BRCA1 [3] (one misclassification of 27 cases; Additional file 1) were ignored by ECD due to our extreme criteria. Similarly, SCGB1A1, identified by The Cancer Gene Database as a cancer-related gene [61], was also filtered out of our gene set due to 100%- correct classification request of our procedure. In addition, among the 26 gene markers identified by Su et al. [37], the MWT identified only 7 genes as potential gene markers. Thus, the stringent 100%-correct classification criteria could be relaxed to 7% misclassification error rate, but to control the false discovery rate we should increase the fold-change cut-off value as well. For the MWT classifier we used 1.35 as the minimum value of fold-change for the 100%-correct classification criteria. We could decrease the cut-off fold-change value down to 1.2, with 89%-correct classification criteria. In these cases, the relaxed criteria provide an increment in the enrichment of the number of differentially expressed genes and potential AC gene markers included in our AC Meta-signature. We still have significantly strong enrichment of the Meta-signature genes in extended gene set (Fisher exact two-tail p-value=9.40E-127 and EF = 2.34). Such conditions allow including the above mentioned genes, as well as many other Meta-signature genes in our extended gene set. More accurate explorations of the discriminative gene signature method will allow development of more statistically- and computationally- oriented feature selection methods.

Another area for improvement could be the use of datasets that are comprehensive and have more homogenous patient populations, such as pre-malignant lesions, tissues at the early stage of tumour progression and patients with different immune status.The human lung tissue QRT-PCR array assay of 24 paired mRNA samples of cancer and adjacent normal lung tissues was used to validate two genes SPP1 and CENPA, which perfectly discriminate lung AC from PT-ATs with consistent fold-changes. These genes are completely discriminative and functionally essential and, thus, could be considered for a simple and accurate diagnostic kit of lung AC.

Many other genes and gene subsets which are included in our ECD signature could be validated using expression data from independent cohorts. For example, immunity signature, defined in this study could be basic set for development early AC diagnostic and prognostic kit. These gene set accomplished 100% discrimination ability of the pairs of normal and lung cancer tissue samples, indicating discovery of many novel biomarkers with a significant potential for clinical applications.

## Conclusions

In many medical applications, analysis of data taken from paired samples of the same patient could increase efficiency of the feature selection and reduce a risk of misclassification. We develop a novel feature selection method of microarray analysis of dependent paired sample design. The method identifies and selects highly discriminative variables from high-dimensional data space based on a statistical analysis of paired samples when the number of samples is small. This method provides superior selection in comparison to conventional methods and can be widely used in different applications.

ECD reveals ~2,300 patho-biologically essential genes associated with the global transcriptional reprogramming of human lung epithelium cells and lung AC aggressiveness. This gene set includes many known prospective AC biomarkers reflecting inherent disease complexity and specifies the mechanisms of carcinogenesis in the lung AC.

The extremely discriminative genes form four contrast gene co-expression clusters with easy biological interpretation. The 1^st^ most strong cluster represented by lung AC mitotic/cell cycle up-regulated genes, 2-nd cluster is represented by normal lung epithelial cells at the strong suppression, 3-rd and 4-th clusters are represented by the genes involved in cell communication, motility and the immune system.

We identified novel immune system signature of lung AC, which might be used for future development of diagnostics, monitoring and prognosis of the patients with AC.

17% of the proteins encoded by the discriminative genes contain known mutagenesis sites. Similar fraction of the known mutagenesis sites was found in the Meta- signature set. We suggest that mutagenesis site-containing proteins found in both of our signatures could be considered as casuistic players in pathobiology of AC and prospective gene markers directly involved in molecular mechanisms driving lung carcinogenesis and progression of AC.

A good agreement of selected genes with the set of >1000 published potential molecular biomarkers and AC signature genes was found. These genes as well as novel genes of ECD-defined lung AC biomarker space could be considered as the comprehensive source of novel biomarkers. The human lung tissue QRT-PCR array assay of 24 paired mRNA samples of cancer and adjacent normal lung tissues was used to validate two AC discriminating genes SPP1 and CENPA. Both of these genes separate the normal and cancerous mRNA expression signals in all 24 pairs studied. We suggest that these genes could be considered as the prospective prognostic and diagnostics biomarkers of the human lung AC.

## Methods

### Dataset

The present study used Su et al. [37] microarray data downloaded from NCBI's GEO database (http://www.ncbi.nlm.nih.gov/geo/), under the GEO accession number GSE7670. The lung tissue samples were obtained during the lung cancer patients’ surgery. The authors selected adjacent normal-tumour matched lung cancer pair samples for RNA extraction and hybridization of mRNA to Affymetrix U133A microarray platform (22,283 probe sets) based on the manufacturer’s standard protocol. In total, 66 microarray samples were available (which also included expression measurements on several lung cancer cell lines, normal lung tissue and mixed tissues). We used 54 pair-wise tissue samples, selected from 27 patients.

The microarray hybridization signal intensity values of the 22,283 U133A probe sets were MAS5.0 calibrated, and the values were scaled by adjusting the mean signal intensity values to a target value of log500. For the present study, we used non-log transformed data (log-transformed data provided slightly worse results; data not presented).

### General model of paired dependence test

The workflow of our discriminative feature selection method is given in Figure 1. Briefly, a feature (probeset or associated RefSeq gene) *j*, *j* (*j* = 1, …, M), measured in the cancer (class1 or Y) and surrounding tissue samples (class2 or X) of N patients, *i* = 1, …, N, is discriminative if there is a significant difference between the expression levels of the two classes. Let  denote the microarray expression signal of patient *i*, probeset j and class Y. Similarly denote as  the paired data of class X. We assume no missing data for each pair pair . The paired dependence test compares the repeated measurements within subjects, rather than across subjects, and in our case has greater power than an unpaired test.

In a classical univariate paired difference analysis, we would first subtract the non-disease value from the disease value for each measured variable of the same subject, then compare these distribution of the differences around zero. Mathematically, let  and  denote the average difference of probeset *j* across the N patients.

The mean difference between the groups does not depend on whether we organize the data as pairs. The mean difference is the same for the paired and unpaired statistics but their statistical significance levels can be very different, because of the estimation of the variance component. Below we show that the variance of D_j_ in paired samples is lower because there is positive correlation within each dependent pair.

### A random effects model for paired testing

The following statistical model is useful for understanding the paired difference test:

*Y_ij_* = μ*_j_* + α*_i_* + ε*_ij_*

where α*_i_* is a random effect that is shared between the two values in the pair, and ε*_ij_* is a random noise term that is independent across all data points. The constant values μ_1_, μ_2_ are the expected values of the two measurements being compared, and the test interest is in δ = μ_2_ − μ_1_.

In this model, the α*_i_* capture "stable confounders" that have the same effect on the “normal” tissue and “abnormal” tissue measurements. In unpaired data analysis the α*_i_* cancels out, so it does not contribute to the variance. In paired data the within-pairs covariance is *Cov*(α)>0 which decreases the variance of δ differences and leads to better performance.

### Modified Wilcoxon test (MWT)

MWT is a simple non-parametric separability test of paired data, which can be used to select the highly discriminative features (gene markers).

Obtaining a sufficiently large number of sample pairs in clinical microarray studies is a very difficult task. Usually, the number of paired samples is smaller than that in studies of independent samples. To cope with these constraints, a nonparametric approach is appropriate. Specifically, the Wilcoxon signed (WT) rank test [62] can be used. This test uses the model(1)

where D*_ij_* has been defined above θ is the unknown ‘disease effect’, and e*_ij_* are random errors, which are assumed to be mutually independent and symmetric about zero.

To test the null hypothesis of no disease effect is equivalent to test:(2)

Our modification of the model of WT (MWT) involves the Cross Normalization step and the Wilcoxon sign rank test as follows:

1: Calculate the class average across samples: .

2: Cross-normalize the average of the opposite class(3)

where  and  are the average expression levels for probeset j across N patients at classes Y and X, respectively.

3: Calculate differences between cross-normalized vectors for each sample pair:(4)

4: Calculate the absolute value of the differences obtained from Step 3.

5: Rank the differences obtained from Step 4 (*R*^-/+^).

6: Obtain the sum of the total ranks of each individual sign (+ and -).

7: Calculate the Z-value using the smaller value *R*^-/+^ (Step 6) with the following formula:(5)

where:(6)

And *N* is the total number of paired samples.

8: Introduce the correction factor of  into the Z-value:(7)

9: Calculate the p-value from the Z-table [62] and disregard data that are less than the defined threshold of 0.05.

10: Calculate the separation error of each feature (gene expression signal):

Let t^+VE^ denote the number of patients that have a positive d_j_.

Let t^-VE^ denote the number of patients that have a negative d_j_

Calculate the percentage of classification errors, by using the smaller value of t^+VE^ and t^-VE^ over N ( or ).

11: Select only the probe-sets with the number of classification errors equal to zero.

Notice: Steps 3 – 7 are used by the original Wilcoxon signed-rank test.

Note using CN procedure, our paired dependence analysis (3) combines the properties of the parametric symmetry (zero mean the differences) test and the non-parametric (zero rank the differences) symmetry test.

### The sign symmetry test

A sign test could be used to estimate the probability of misclassifications error in paired sample experimental design (tumour vs. normal tissues from the same organ of a patient). For each microarray expression signal i, given the gene expression intensity values of 27 independent patients measured in two conditions, we can estimate the probability of observing less than s patients' misclassifications. This probability of observing an event that is more extreme than s (p-value) and can be estimated by the Binomial distribution. Assuming that the probability of positive and negative increments is p = 0.5 the probability of misclassification is the following:

For example the probability of at most two misclassifications in 27 paired samples is calculated as:

which after Bonferroni correction on the number of U133A probe sets becomes adjusted p-value = 0.062.

### A bootstrap test of stochastic equality of two populations

Bootstrapping testing [63] was used to calculate the significance of each Affymetrix U133A probe set hybridization signal. To calculate the bootstrap P value the samples were randomly resampled (with replacement) 9,999 times, MWT was run and Pboot was estimated. The P values of interest is Bootstrap P = (number of times Pboot < Ptest)/9999. Only the signals with bootstrap p-values less than 0.05 were considered.

### Programming based on MWT

A program in C++ language was written for the MWT method to process the microarray Affymetrix data sets. The data, when processed through the program, generate an N x M table of cross-normalized data, where M is the number of the Affymetrix ID and N is the number of the Patient’s ID. Another N x M table contains 1-s and 0-s, where 1 represents positive signs and 0 represents negative signs of gene expression in the pair. This indicates the relative down- or up- regulation of a given hybridisation signal (corresponding to the expression level of the gene transcript) for a given object of the class Y (cancer tissue) in comparison to the paired object of the class X (normal tissue). Finally, the M U133A probe set IDs (representing the M columns of the table) are sorted based on the MWT p-values, and the total number of 1-s (and percentage) for each microarray probe set ID is tabulated. The program also calculates the ratio of the average expression signals of Class 2 to Class 1.

### Hypergeometric P-Value

Hypergeometric function (Fisher exact) test [54,62] is use to assess the significance of the overlapping region in the Venn diagram. The terms we use are as follows:

*k*: Number of elements in the overlapped region of the Venn diagram

*n*: Total number of elements present in group 1

*m*: Total number of elements present in group 2

*N*: Total number of elements present in both groups.

To find the probability that *k* or more elements intersect subsets of *n* and *m* members at random (or the p-value for overlap of *k*) in a universe set of size *N*, we summon over the right tail of a hypergeometric distribution:

Pr(*k* ≥ *l ; n*, *m*, *N*) := *p-value*

This is a one-sided test where the p-values correspond to over-represented lists of elements (genes) in the intersection region of the Venn diagrams. In the case of Refseq genes non-redundantly presented on U133A microarray *N* equals 13074.

### Student’s t-test and Wilcoxon test with FDR correction

The standard Student’s t-test for paired data and the non-parametric Wilcoxon signed-rank test for paired data, implemented in the R statistical package as the ‘t.test’ and ‘wilcox.test’ functions, were used to compare the expression levels in pairs of normal and tumour samples. The resulting P-values were corrected with the FDR values using p-value adjustment module ‘p.adjust’, implemented in the R statistical package. The ensuing FDR values were used to rank the genes, and the desired number of genes was then selected for use.

### EDGE analysis

An optimal discovery procedure (ODP) method, implemented in the Extraction of Differential Gene Expression (EDGE) [64] program, was used to assess the significance of the differential expression of the genes between paired tumour and normal tissue samples of the same patients. The paired microarray information was incorporated into EDGE, using the ‘MATCHED DESIGN SETTINGS’ option. The false discovery rate (FDR) for selected genes was monitored and controlled by calculating the q-value. By default, for the present dataset, genes with a q value <0.00021 were considered as significantly differentially expressed genes between the comparison groups by EDGE. However, to allow comparison with other methods, the genes were rank ordered using the FDR values, and the desired numbers of genes were then selected for further analysis.

### PAM and SAM analysis

Initially, we ran the PAM algorithm [51] with all 22,283 U133A probe sets as input in the tumour to normal sample comparisons, and acquired an extreme discrimination set of 77 probe sets, which gave the lowest misclassification (error) rate: 0 of 27 for normal and 1 of 27 for tumour predictions. The paired array information was incorporated into PAM by using identical sample IDs for the paired normal-tumour samples. SAM analysis [50] was also used for selection of differentially expressed gene signals.

### Hierarchical cluster analysis

Hierarchical cluster analysis was performed with XCluster [65], using both Euclidian- and centred Pearson correlation coefficient-distance metrics and average linkage to measure the cluster distances during partitioning. Two-way clustering was employed to group both microarrays and samples on one axis and the probe sets on the other axis. The resulting clustered structures were displayed as heatmaps, using the Java Tree View software [66].

### GO analysis

Diavid Bioinformatics [67], ClueGO [68], Panther Bioinformatics [69] and GeneGo(Metacore) [70] tools were used to evaluate the Gene Ontology terms enrichment in gene/protein groups.

### Tissue scan qRT-PCR array

Primers were designed for validating 2 discriminative genes which perfectly classify lung tumours from surrounding normal tissues (obtained based on computational methods). Relative levels of mRNA of genes selected for validation were quantified using Tissue scan panel HLRT-02 which includes 24 lung cancer cDNA samples matched in pairs with surrounding lung (normal) cDNA samples. This panel was provided by the OriGene Technologies, Rockville MD (manufacture). The panel contains pre-normalized 48 cDNA samples with various subtypes of lung cancer with information regarding TNM, tumour size, tumor type, stage, age and other clinical information. qRT-PCR experiments were carried out on Applied Biosystems 7500 Real Time PCR machine. The normal to tumour Ct-values of each gene were obtained and relatively quantified using ΔΔCt analysis [71]. Primers were designed for SPP1 (For- CTC CAT TGA CTC GAA CGA CTC and REV- CAG GTC TGC GAA ACT TCT TAG AT ) and CENPA (For- AGC ACA CAC CTC TTG ATA AGG A and REV- CAC ACC ACG AGT GAA TTT AAC AC ) along with control Actin-B (commercially provided).

## Competing interests

The authors declare that they have no competing interests.

## Authors' contributions

The conception of analysis strategies was contextualized by VAK. He suggested the theory of the Extreme Class-Discrimination method and the strategy of the performing the bioinformatics and statistical analyses of lung adenocarcinoma data. SHT and PP were in charge of major part of the computational calculations and the statistical data analysis and involved in the generation of the specialized computer programs used in this work. They also were participated in writing the draft of the manuscript. EM was involved in the discussion of the statistical methods, the interpretation of the results and in manuscript correction and revision. SPY provided PCR validations of the prospective biomarkers and discussed the results of this study. KCK was involved in discussions of this work. VAK did data analysis and conceived the study, coordinated the project, and wrote the final version of the manuscript.

## Supplementary Material

Additional file 1 **Table S1.** Master table annotated with gene symbols with gene symbols from Lung AC meta gene signatures, default and relaxed lung AC gene signatures and signatures derived by other methods cited in the literature.A: Characteristics of the data set and final results; B: Read me File C: Summary statistics. D: AC signature derived by SAM software from U95A data. S1e: Tables of the 604-gene PT-AT consensus signature, the results of GO analysis, the immunity and cell cycle gene signatures discriminating AC from normal lung tissues. Click here for file

Additional file 2 **Table S2.** Distribution of the number of false classifications of the 27 paired samples. Comparison of the classification accuracy of the extremely discriminative 2,829 probe sets (ECD) with the top-level 2,829 probe sets identified by the standard Wilcoxon sign ranked test (WT), EDGE, PAM, and Student’s t-test. (ECD signature was selected using MWT on cross-normalized signal intensities with 100% accuracy criteria and a bootstrap p-value cut-off<0.05). While the ECD classifier was derived using the cross-normalized dataset as input, the classifiers derived using PAM, EDGE, WT and t-test used the original MAS5-normalized data as input. However, the classification accuracy, in terms of the number of probe sets with 2 or more anomalous fold-changes, was estimated using the MAS5-normalized dataset. Click here for file

Additional file 3 **Comparison of different feature selection methods. Analysis of discriminative ability using the original MAS5 normalized data** The two-way hierarchical cluster analysis of the 2,829 probe sets and the 27 pairs of normal-lung AC samples demonstrates the ability of the selected methods to separate lung AC from normal samples (Supplementary figures S1-S4). All of the methods, with the exception of the t-test and the Limma paired test, produced a near-perfect separation of the two classes, however, ECD provides more biologically reasonable grouping of the genes (see Results). Figure S1. Two-way hierarchical cluster analysis of the MAS5-normalized expression values of 2,829 probe sets identified by the standard Wilcoxon test. Figure S2. Two-way hierarchical cluster analysis of the cross-normalized expression values of the 2,829 probe sets identified by EDGE. Figure S3. Two-way hierarchical cluster analyses of (A) the MAS-normalized expression values and (B) the cross-normalized expression values of 2,829 probe sets identified using the Student’s t-test. Figure S4. Two-way hierarchical cluster analyses of (A) the MAS-normalized expression values and (B) the cross-normalized expression values of 2,829 probe sets identified using the Limma paired t-test (Smyth, G. K., 2005). Click here for file

Additional file 4 **Table S3.** Meta-gene signatures associated with Lung AC, curated from the literature with the corresponding references. Click here for file
