# Supplementary material for: A robust tool for discriminative analysis and feature selection in paired samples impacts the identification of the genes essential for reprogramming lung tissue to adenocarcinoma
Source: BMC Genomics. 2011 Nov 30;12(Suppl 3):S24. doi: 10.1186/1471-2164-12-S3-S24 (PMC3377915; doi:10.1186/1471-2164-12-S3-S24)
Supplement: Additional file 2 — Table S2. Distribution of the number of false classifications of the 27 paired samples. Comparison of the classification accuracy of the extremely discriminative 2,829 probe sets (ECD) with the top-level 2,829 probe sets identified by the standard Wilcoxon sign ranked test (WT), EDGE, PAM, and Student’s t-test. (ECD signature was selected using MWT on cross-normalized signal intensities with 100% accuracy criteria and a bootstrap p-value cut-off<0.05). While the ECD classifier was derived using the cross-normalized dataset as input, the classifiers derived using PAM, EDGE, WT and t-test used the original MAS5-normalized data as input. However, the classification accuracy, in terms of the number of probe sets with 2 or more anomalous fold-changes, was estimated using the MAS5-normalized dataset. [file 1471-2164-12-S3-S24-S2.pdf]

Table S2. Distribution of the number of false classifications of the 27 paired samples.

A. Comparison of the classification accuracy of the extremely discriminative 2,829 probe sets (ECD classifier) with the top-level 2,829 probe sets identified by the standard Wilcoxon sign ranked test (WT), EDGE, PAM, and Student's t-test. (ECD classifier was selected using MWT on cross-normalized signal intensities with 100% accuracy criteria and a bootstrap p-value cut-off<0.05). While the ECD classifier was derived using the cross-normalized dataset as input, the classifiers derived using PAM, EDGE, WT and t-test used the original MAS5-normalized data as input. However, the classification accuracy, in terms of the number of probe sets with 2 or more anomalous fold-changes, was estimated using the MAS5-normalized dataset.

	False Classifications	EDGE	PAM	Wilcoxon test	Student t-test
UP regulated in tumors	0	43	40	43	43
	1	154	136	154	153
	2	262	211	268	261
	3	285	207	296	286
	4	275	193	278	269
	5	188	178	152	176
	6	98	151	60	80
	7	29	81	5	22
	8	6	38	0	2
	9	0	16	0	0
	10	0	3	0	0
	11	0	1	0	0
	12	0	0	0	0
	13	0	1	0	0
UP regulated in normal	13	0	0	0	0
	12	0	0	0	0
	11	0	3	0	0
	10	0	2	0	0
	9	0	11	0	0
	8	4	35	1	3
	7	18	51	12	19
	6	53	117	56	63
	5	122	149	141	134
	4	228	207	264	241
	3	276	247	299	285
	2	314	287	323	317
	1	302	294	305	303
	0	172	171	172	172
# of genes with 2 or more errors		2158	2188	2155	2158
Total # of genes with		2829	2829	2829	2829
percent of genes with 2 or more errors		0.762	0.773	0.762	0.763

B. Comparison of significance p-values of common GO terms (Biological process) obtained after feature selection by the ECD (MWT) and EDGE methods from 27 paired Affymetrix U133A AC-AT arrays data

	EDGE	MWT
GOTERM_BP_FAT	P-Value	P-Value
cell cycle	4.40E-04	1.50E-08
mitotic cell cycle	9.20E-05	1.00E-08
cellular macromolecular complex assembly	3.20E-02	9.00E-06
cellular macromolecular complex subunit organization	2.20E-02	1.60E-05
cell cycle process	2.90E-04	9.00E-07
nucleosome organization	3.40E-02	2.00E-04
chromatin assembly	2.30E-02	2.30E-04
DNA packaging	1.00E-02	1.10E-04
macromolecular complex subunit organization	3.20E-02	3.70E-04
regulation of cell cycle	9.00E-03	1.10E-04
cell death	3.80E-02	5.70E-04
nucleosome assembly	1.90E-02	4.30E-04
death	2.80E-02	7.30E-04
regulation of cell migration	3.20E-02	1.20E-03
regulation of cellular protein metabolic process	3.10E-02	1.20E-03
protein amino acid O-linked glycosylation	2.90E-02	1.20E-03
regulation of cell motion	4.40E-02	2.30E-03
chromatin assembly or disassembly	4.10E-02	2.20E-03
