# Supplementary material for: A robust tool for discriminative analysis and feature selection in paired samples impacts the identification of the genes essential for reprogramming lung tissue to adenocarcinoma
Source: BMC Genomics. 2011 Nov 30;12(Suppl 3):S24. doi: 10.1186/1471-2164-12-S3-S24 (PMC3377915; doi:10.1186/1471-2164-12-S3-S24)
Supplement: Additional file 3 — Comparison of different feature selection methods. Analysis of discriminative ability using the original MAS5 normalized data The two-way hierarchical cluster analysis of the 2,829 probe sets and the 27 pairs of normal-lung AC samples demonstrates the ability of the selected methods to separate lung AC from normal samples (Supplementary figures S1-S4). All of the methods, with the exception of the t-test and the Limma paired test, produced a near-perfect separation of the two classes, however, ECD provides more biologically reasonable grouping of the genes (see Results). Figure S1. Two-way hierarchical cluster analysis of the MAS5-normalized expression values of 2,829 probe sets identified by the standard Wilcoxon test. Figure S2. Two-way hierarchical cluster analysis of the cross-normalized expression values of the 2,829 probe sets identified by EDGE. Figure S3. Two-way hierarchical cluster analyses of (A) the MAS-normalized expression values and (B) the cross-normalized expression values of 2,829 probe sets identified using the Student’s t-test. Figure S4. Two-way hierarchical cluster analyses of (A) the MAS-normalized expression values and (B) the cross-normalized expression values of 2,829 probe sets identified using the Limma paired t-test (Smyth, G. K., 2005). [file 1471-2164-12-S3-S24-S3.pdf]

Additional file 3 (*.pdf)

Figures S1-S4. Comparison of different feature selection methods

Analysis of discriminative ability using the original MAS5 normalized data

The two-way hierarchical cluster analysis of the 2,829 probe sets and the 27 pairs of normal-lung AC samples demonstrates the ability of the selected methods to separate lung AC from normal samples (Supplementary figures S1-S4). All of the methods, with the exception of the t-test and the Limma paired test, produced a near-perfect separation of the two classes, however, ECD provides more biologically reasonable grouping of the genes (see Results).

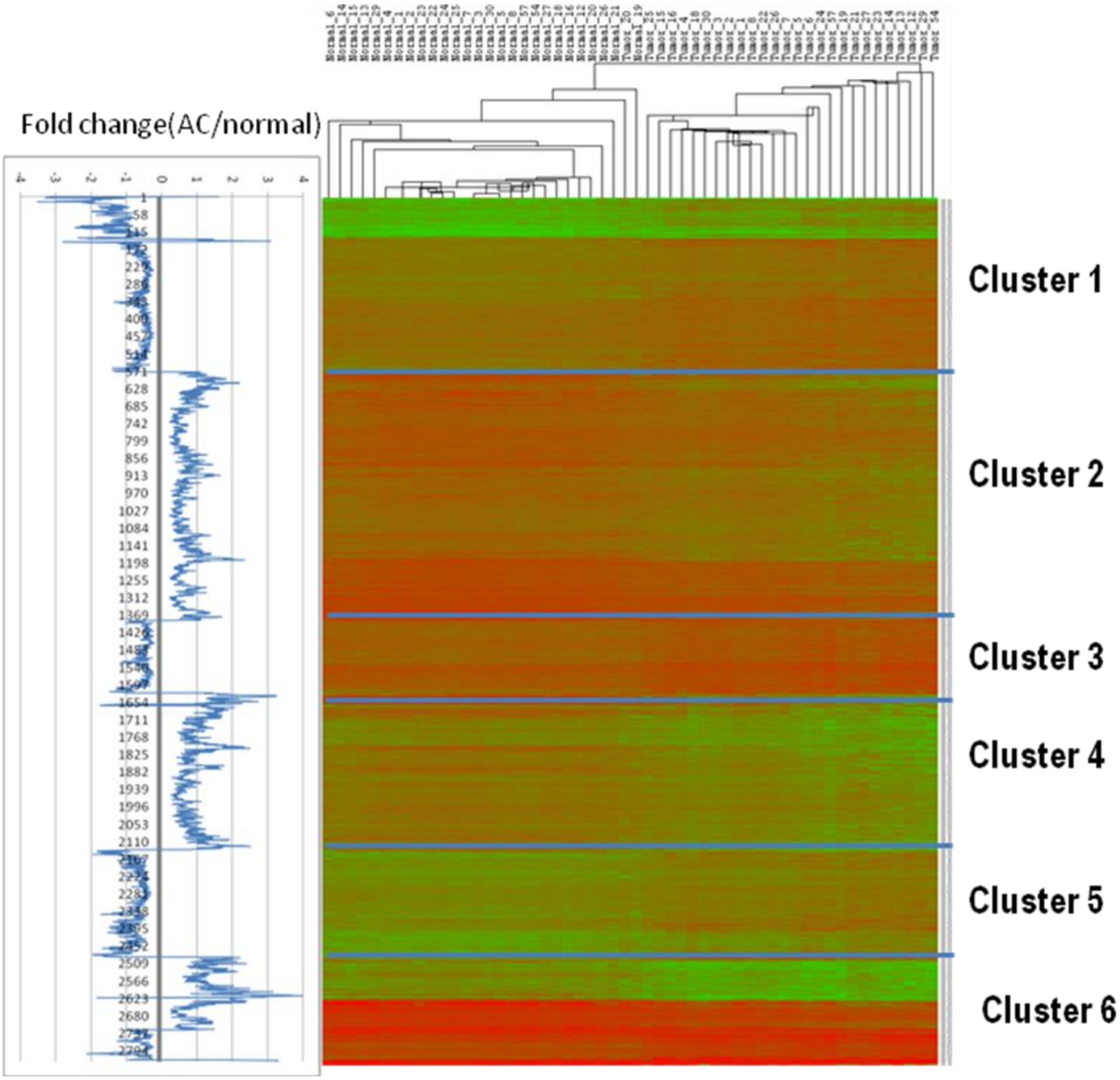

Figure S1. Two-way hierarchical cluster analysis of the initial MAS5-normalized expression values 2829 probsets identified by standard Wilcoxon sign ranked test. 6 gene clusters are found, however they does not associated with grouping of the patients.

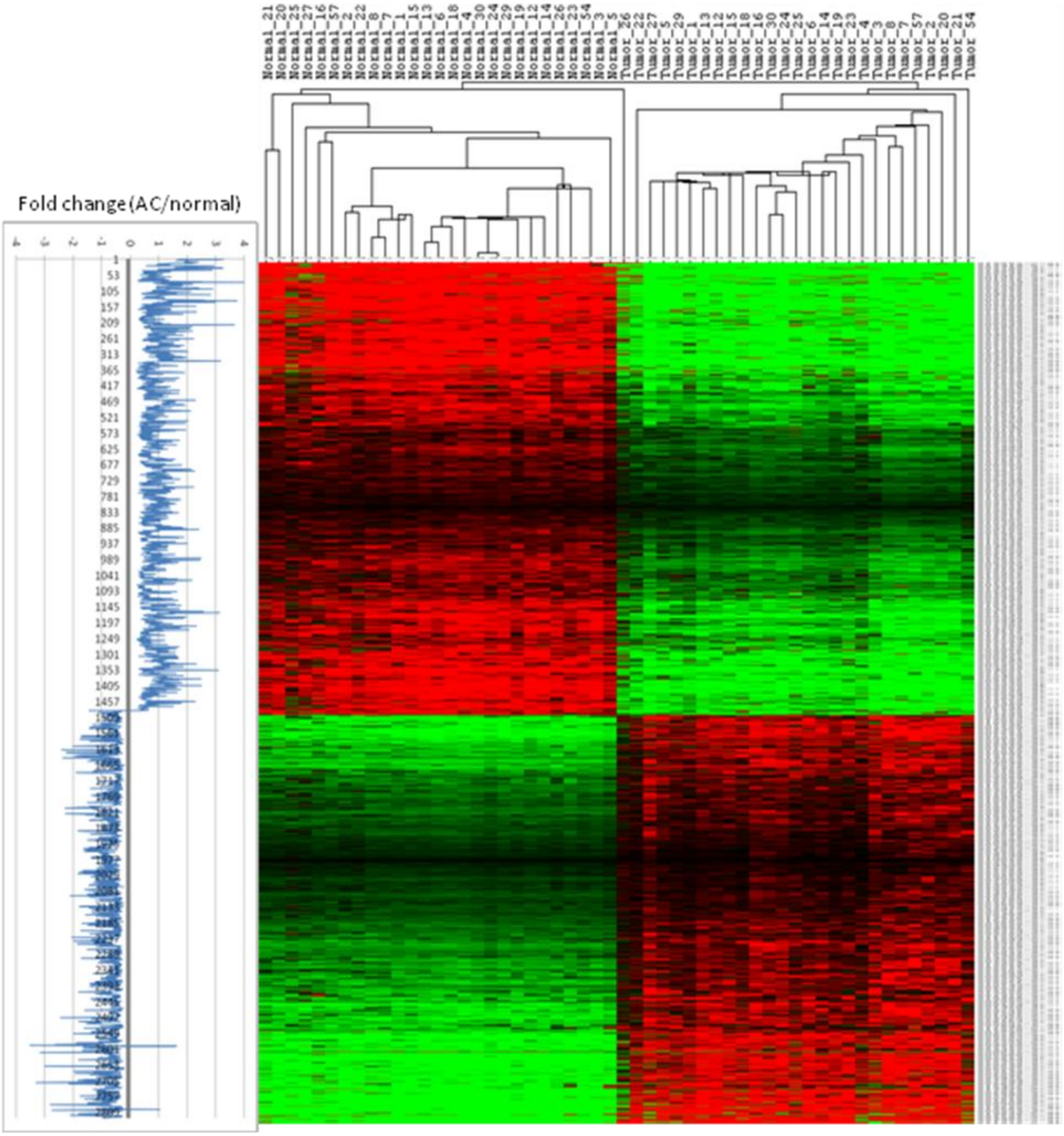

Figure S2. Two way hierarchical cluster analysis of the cross-normalized expression values of the 2829 probsets identified by EDGE. Two gene clusters are represent AC and AT respectively.

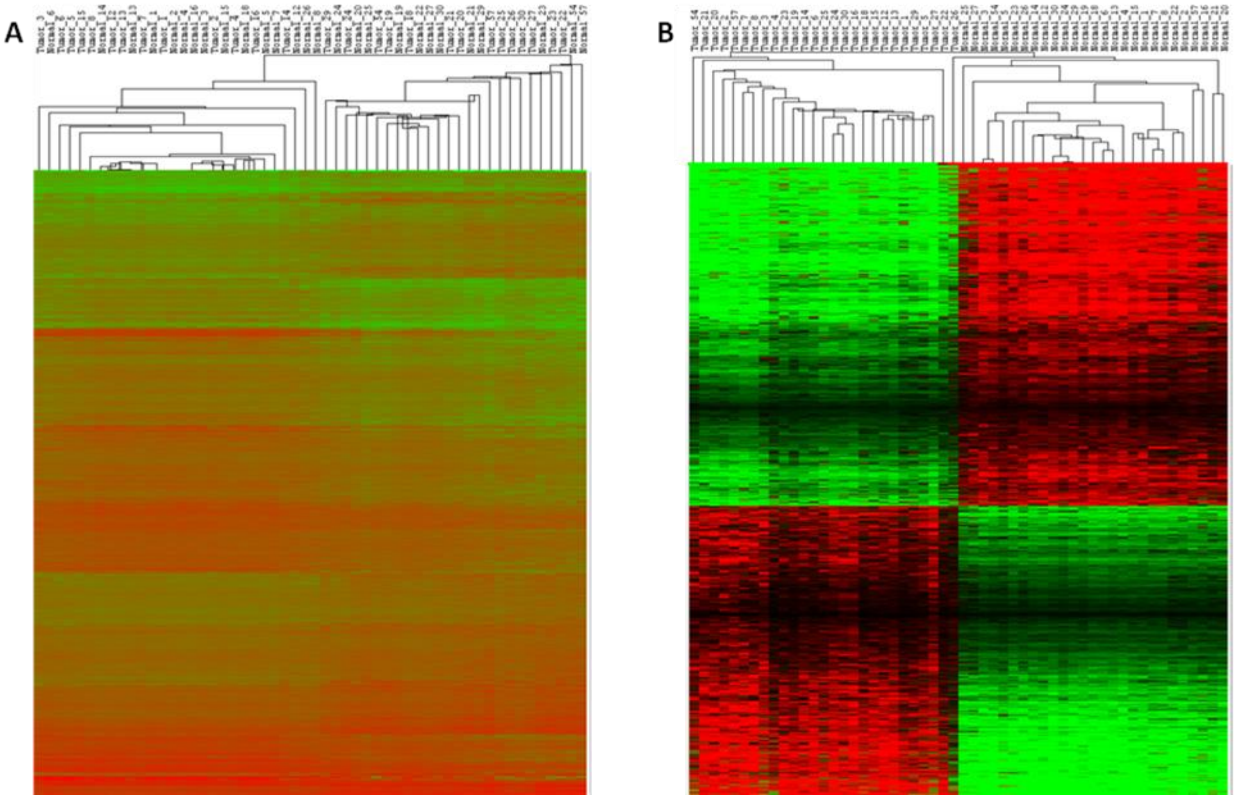

Figure S3. Two way hierarchical cluster analyses of (A) the initial MAS-normalized expression values and (B) the cross-normalized expression values of 2829 probesets identified using Student t-test.

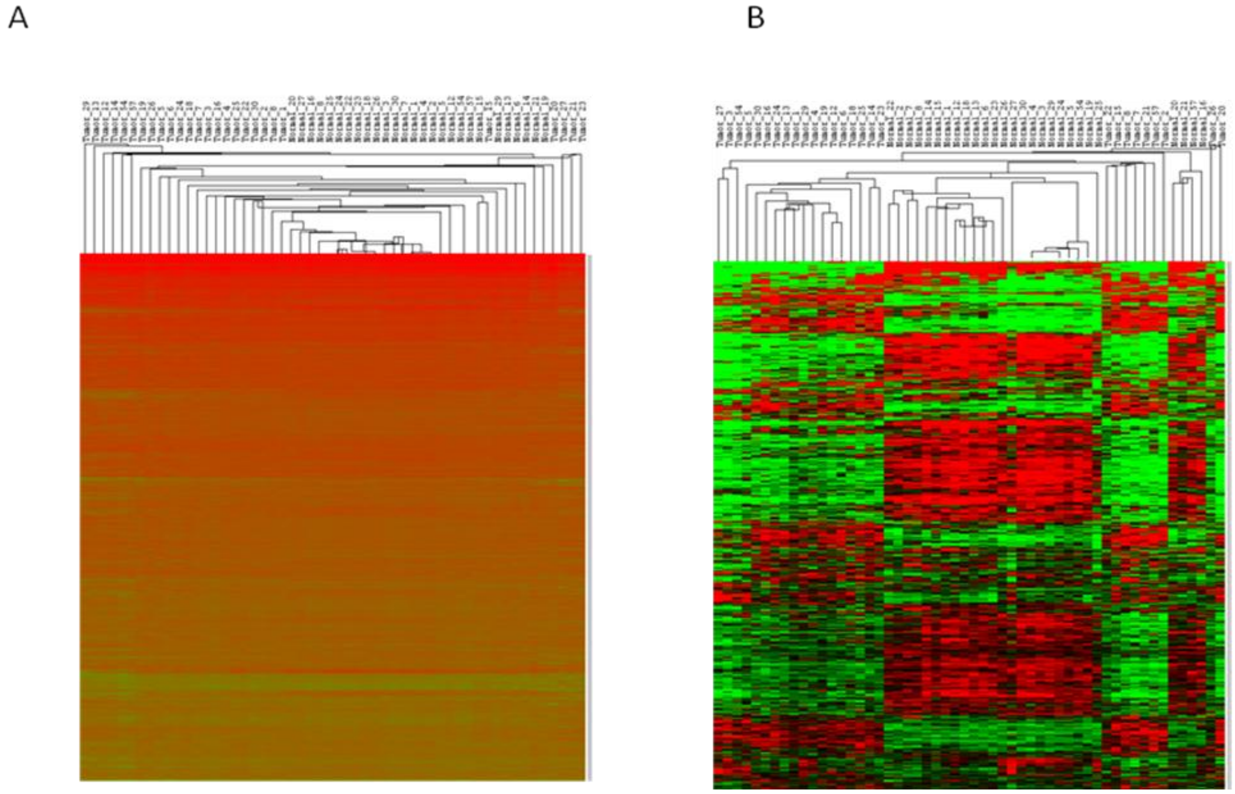

Figure S4. Two way hierarchical cluster analyses of (A) the MAS-normalized expression values and (B) the cross-normalized expression values of 2829 probesets identified using Limma paired t-test (*). Probsets, selection by this test is failed to clustering the genes and patients.

* Smyth, G. K. (2005). Limma: linear models for microarray data. In: Bioinformatics and Computational Biology Solutions using R and Bioconductor, R. Gentleman, V. Carey, S. Dudoit, R. Irizarry, W. Huber (eds.), Springer, New York, pages 397–420.
