# Supplementary material for: A robust tool for discriminative analysis and feature selection in paired samples impacts the identification of the genes essential for reprogramming lung tissue to adenocarcinoma
Source: BMC Genomics. 2011 Nov 30;12(Suppl 3):S24. doi: 10.1186/1471-2164-12-S3-S24 (PMC3377915; doi:10.1186/1471-2164-12-S3-S24)
Supplement: Additional file 4 — Table S3. Meta-gene signatures associated with Lung AC, curated from the literature with the corresponding references. [file 1471-2164-12-S3-S24-S4.pdf]

Table S4. Meta-gene signatures associated with Lung AC, curated from the literature with the corresponding references[#]

Gene Cards ID	Ref[#]	no U133A probesets
AADAC	[3]	
ABAT	[5]	
ABCA2	[12]	
ABCA4	[3]	
ABCC1	[12]	
ABCC3	[3]	
ABCC6	[5]	
ABI2	[5] [12]	
ABL2	[11]	
ABLIM1	[3]	
ACACB	[5]	
ACO2	[25]	
ACOT8	[13]	
ACSS3	[13]	
ACTB	[3]	
ACTL6A	[5]	
ACTL6B	[25]	
ACTR2	[14]	
ACVR1B	[11]	
ACVRL1	[11]	
ADAM17	[12]	
ADAM8	[3]	
ADAMTS1	[14]	
ADAMTSL2	[13]	
ADCY9	[5]	
ADH1A	[2]	
ADH1B	[3]	
ADH1C	[9]	
ADIPOR2	[3]	
ADM	[4]	
ADRA2C	[13]	
AGER	[9]	
AGFG1	[4]	
AGR2	[1]	
AGTR2	[14]	
AHNAK	[3]	

Gene Cards ID	Ref[#]	no U133A probesets
AIM1L	[25]	
AKAP12	[4]	
AKAP13	[2]	
ALDH1A1	[22]	
ALDH3B2	[4]	
ALDH9A1	[22]	
ALDOA	[4]	
ALOX5	[3]	
ALOX5AP	[3]	
AMFR	[22]	
ANGEL2	[5]	
ANK2	[14]	
ANKHD1	[5]	
ANKHD1-EIF4EBP3	[5]	
ANKRD49	[5]	
ANXA3	[3]	
ANXA8	[3]	
AOAH	[13]	
AOC3	[3]	
AP1G2	[3]	
AP1S1	[25]	
AP3S2	[5]	
AP4M1	[25]	
APC	[12] [11]	
APOE	[3]	
APOL6	[14]	
AQP3	[3]	
AQP4	[14]	
AQP5	[3]	
ARAF	[11]	
ARFGEF2	[3]	
ARHGAP11A	[5]	
ARHGAP8	[3]	
ARHGDIB	[22]	

Gene Cards ID	Ref[#]	no U133A probesets
ARHGDIG	[2]	
ARHGEF1	[12]	
ARHGEF10	[3]	
ARHGEF2	[2]	
ARID5A	[5]	
ARL4A	[12]	
ARPC2	[3]	
ASAH1	[13]	
ASF1B	[5]	
ASPM	[5]	
ATAD2	[5]	
ATM	[11]	
ATP10B	[7]	
ATP13A3	[5]	
ATP1B1	[3]	
ATP2B1	[5] [4]	
ATP5A1	[2]	
ATRX	[2]	
AURKA	[5] [7]	
AURKB	[5]	
AZGP1	[14] [3]	
B4GALNT1	[12]	
BAG1	[4]	
BAP1	[11] [3]	
BAX	[11]	
BBC3	[9]	
BBS9	[4]	
BCAM	[5] [9]	
BCL2	[12]	
BIK	[12]	
BIRC5	[5]	
BLCAP	[25]	
BLM	[5] [12]	
BMP2	[9] [4]	
BNIP1	[12]	

Gene Cards ID	Ref#	no U133A probesets
BRCA1	[18]	
BRCA2	[5]	
BRD9	[25]	
BRIP1	[5]	
BTG2	[14]	
BTK	[22]	
BUB1	[5]	
BUB1B	[5]	
BZW1	[9] [4]	
C10orf116	[3]	
C11orf17	[5]	
C12orf48	[5]	
C13orf27	[5]	
C14orf132	[3]	
C16orf59	[5]	
C19orf57	[13]	
C1orf112	[5]	
C1orf54	[5]	
C1QTNF3	[13]	
C1S	[14]	
C20orf46	[13]	
C21orf33	[12]	
C2orf3	[5]	
C2orf34	[5]	
C4A	[3]	
C4A C4B	[5]	
C4BPA	[3]	
C4orf10	[12]	
C6orf105	[5]	
CA4	[3]	
CACNA1D	[5]	
CACNA2D2	[11]	
CACNB1	[3]	
CACNG2	[25]	
CAD	[5]	
CADM1	[5]	
CALCRL	[3]	
CAMK1G	[5]	
CAMKK2	[14]	

Gene Cards ID	Ref#	no U133A probesets
CAPNS1	[3]	
CAPRIN1	[5]	
CAPZA2	[3]	
CASP10	[12]	
CASP4	[4]	
CASP8	[12]	
CAT	[3]	
CAV1	[3]	
CAV2	[3]	
CBLC	[16]	
CBX3	[5] [12]	
CCDC21	[25]	
CCDC85B	[3]	
CCL11	[25]	
CCL19	[3]	
CCL2	[25]	
CCL20	[14]	
CCL7	[25]	
CCL8	[25]	
CCNA2	[5]	
CCNB1	[5] [2]	
CCNE2	[5]	
CCPG1	[5]	
CCR2	[12]	
CCR7	[11] [9]	
CCT5	[5]	
CD24	[15] [3]	
CD27	[5] [3]	
CD38	[13]	
CD48	[13]	
CD52	[3]	
CD52	[25]	
CD53	[13]	
CD79A	[3]	
CD8B	[2]	
CDC20	[5]	
CDC25A	[5]	
CDC25C	[5]	
CDC42BPA	[11]	
CDC6	[5] [4]	

Gene Cards ID	Ref#	no U133A probesets
CDC7	[5]	
CDCA3	[5]	
CDCA8	[5]	
CDH1	[1]	
CDH11	[11]	
CDH3	[1]	
CDH8	[12]	
CDK13	[12]	
CDK20	[13]	
CDK7	[18]	
CDKN1C	[3]	
CDKN2A	[11]	
CDKN3	[5]	
CDS1	[9] [4]	
CDT1	[5]	
CEACAM5	[13] [3]	
CEACAM6	[3] [1]	
CELF2	[12]	
CENPA	[5]	
CENPE	[5]	
CENPF	[5]	
CENPI	[5]	
CENPM	[25]	
CEP55	[5]	
CEP72	[25]	
CEP76	[5]	
CES1	[3]	
CFD	[9]	
CFHR2	[2]	
CHAF1A	[5]	
CHD4	[2]	
CHEK1	[5]	
CHERP	[12]	
CHI3L1	[3]	
CHIT1	[5]	
CHN1	[3]	
CHPF	[3]	
CHRNA2	[12]	
CIT	[3]	

Gene Cards ID	Ref#	no U133A probesets
CKAP2	[5]	
CKAP4	[4] [9] [2]	
CKAP5	[5]	
CKS1B	[5]	
CKS2	[5]	
CLCC1	[5]	
CLDN3	[3]	
CLDN4	[3]	
CLDN5	[7]	
CLDN7	[1]	
CLEC4E	[13]	
CLIC4	[3]	
CMAH	[5]	
CNGB1	[5]	
CNIH3	[13]	
CNKSR1	[5]	
CNN3	[22]	
COL11A1	[10] [7]	
COL1A1	[3] [2]	
COL4A3	[5]	
COL9A2	[5]	
COPS6	[25]	
CORO1A	[22]	
COX7A1	[9] [3]	
CP	[10] [3]	
CPT1B	[3]	
CRABP1	[12]	
CRABP2	[3]	
CRBN	[5]	
CRCT1	[5]	
CREB3	[2]	
CREB3L1	[3]	
CRK	[4]	
CRTC1	[14]	
CRYL1	[5]	
CRYM	[10] [2]	
CSDA	[3]	
CSDC2	[25]	
CSDE1	[25]	
CSE1L	[5]	

Gene Cards ID	Ref#	no U133A probesets
CSF2RB	[25]	
CSRP1	[5]	
CST6	[3]	
CSTB	[4]	
CTNNA1	[14]	
CTRB1	[9]	
CTSF	[13]	
CTSH	[9]	
CTSL1	[4]	
CTSL2	[5]	
CUL4B	[5]	
CXCL12	[22]	
CXCL3	[22]	
CXCR2	[12]	
CXorf56	[14]	
CYP1A1	[14]	
CYP24A1	[16] [4]	
CYP2B6	[5] [3]	
CYP2B7P1	[5]	
CYP2D6	[5]	
CYTH1	[14]	
CYTIP	[13]	
DAXX	[22]	
DBF4	[5]	
DBH	[12]	
DBP	[22] [4]	
DCK	[5]	
DCLRE1B	[25]	
DDR1	[11]	
DDX11	[5]	
DDX12	[5]	
DDX39	[5]	
DDX3X	[14]	
DEFB1	[4]	
DEK	[5]	
DENND3	[3]	
DEPDC1	[5]	
DIO2	[2]	
DKC1	[5]	

Gene Cards ID	Ref#	no U133A probesets
DKFZp434H1419	[5]	
DLC1	[5]	
DLGAP5	[5]	
DMD	[5]	
DNAI2	[7]	
DNAJB4	[5]	
DNAJC9	[5]	
DNALI1	[5]	
DNMT1	[5]	
DOCK3	[12] [11]	
DOCK4	[3]	
DONSON	[5]	
DPH1	[12]	
DPP4	[3]	
DPYSL2	[3]	
DSCC1	[5]	
DSN1	[5]	
DSP	[12]	
DTL	[5]	
DTNA	[12]	
DTYMK	[5]	
DUSP1	[14]	
DYRK1A	[12]	
E2F1	[5] [18]	
E2F3	[5]	
E2F4	[2]	
E2F8	[5]	
EAF2	[13]	
EBNA1BP2	[2]	
ECT2	[5]	
EDIL3	[5]	
EDNRB	[9]	
EGF	[2]	
EGFR	[11] [3]	
EHD1	[3]	
EHMT2	[18]	
EIF1	[14] [4]	
ELANE	[2]	
ELF3	[1]	

Gene Cards ID	Ref#	no U133A probesets
ELMO1	[3]	
EMP2	[9]	
EMP3	[3]	
ENG	[14] [3]	
ENPP2	[12] [3]	
ENPP4	[5]	
ENSG00000215328	[5]	
EP300	[25]	
EPAS1	[14] [8] [3]	
EPB41L3	[3]	
EPCAM	[1]	
EPHA2	[11]	
EPHA3	[11]	
EPHA5	[11]	
EPHA7	[11]	
EPHB1	[11]	
EPHX1	[3]	
EPHX2	[5]	
EPO	[25]	
EPOR	[5]	
ERBB2	[11] [4]	
ERBB3	[3]	
ERBB4	[11]	
ERCC6L	[5]	
ERMP1	[5]	
ERO1L	[5]	
ESPL1	[5]	
ESYT1	[3]	
ETV6	[12]	
EVL	[5]	
EXOC3	[25]	
EXOSC8	[5]	
EXT2	[13]	
EZH2	[5]	
EZR	[14]	
F11R	[1]	
FABP4	[9] [3]	
FABP5	[3]	
FABP6	[7]	

Gene Cards ID	Ref#	no U133A probesets
FADD	[4]	
FADS3	[3]	
FAM107A	[3]	
FAM129A	[13]	
FAM164A	[5]	
FAM8A1	[5]	
FANCI	[5]	
FBLN1	[3]	
FBN2	[12]	
FBXL12	[25]	
FBXO24	[25]	
FBXO3	[5]	
FBXO4	[5]	
FBXO5	[5]	
FCGBP	[5] [3] [2]	
FCGR3A	[3]	
FCGRT	[9]	
FCN2	[2]	
FCN3	[3]	
FEN1	[5]	
FER	[14]	
FERMT2	[3]	
FEZ2	[4]	
FGF18	[5]	
FGFR4	[11] [3]	
FGG	[14]	
FGR	[3]	
FHL1	[9] [3]	
FIG4	[22]	
FKBP1B	[3]	
FKBP5	[14]	
FKBP8	[3]	
FKBP9	[13]	
FLT3	[12]	
FLT4	[11]	
FMO2	[3] [2]	
FMO5	[3]	
FOLH1	[14]	
FOLR1	[3]	
FOSL1	[5]	

Gene Cards ID	Ref#	no U133A probesets
FOSL2	[5]	
FOXE3	[5]	
FOXF1	[3]	
FOXM1	[5]	
FUCA1	[22] [12] [4]	
FUS	[14]	
FUT1	[3]	
FUT3	[22] [4]	
FUT7	[2]	
FXYD3	[4]	
FYCO1	[5]	
FYN	[11]	
FZD2	[5]	
GABARAPL2	[5]	
GABRA3	[12]	
GABRE	[3]	
GAP43	[22]	
GAPDH	[5] [9] [4]	
GARS	[9] [4]	
GAS2L1	[3]	
GATA2	[3]	
GBP2	[2]	
GCLM	[5]	
GCNT1	[4]	
GDF10	[3]	
GDF15	[3]	
GGCX	[12]	
GHRH	[25]	
GHRHR	[2]	
GINS1	[5]	
GJA4	[3]	
GLB1L2	[3]	
GLG1	[12]	
GLI2	[12]	
GLUD1	[3]	
GM2A	[12]	
GMFG	[3]	
GMPS	[5]	
GNAI2	[3]	

Gene Cards ID	Ref[#]	no U133A probesets
GNAS	[11]	
GNAT2	[12]	
GNB1	[2]	
GNB2	[25]	
GNG11	[3]	
GNG7	[5]	
GNPTAB	[13]	
GNRH1	[12]	
GOLGA1	[12]	
GPC3	[22] [9] [3]	
GPER	[5]	
GPI	[9]	
GPLD1	[12]	
GPR110	[14]	
GPR19	[5]	
GPR6	[2]	
GPRC5A	[3]	
GPX2	[1]	
GPX3	[3]	
GRB2	[5]	
GRB7	[4] [3] [1]	
GRK5	[3]	
GSTO1	[3]	
GSTT2	[13]	
GTF2H2	[22]	
GTF2I	[12]	
GTSE1	[5]	
GUCA2B	[2]	
H1FX	[9]	
H2AFX	[5]	
H2AFY2	[25]	
H2AFZ	[5] [9] [4]	
HABP2	[10]	
HAS1	[14]	
HAUS6	[5]	
HCFC1R1	[4]	
HCG4P5	[5]	
HCG4P6	[5]	
HCK	[11]	

Gene Cards ID	Ref[#]	no U133A probesets
HDAC2	[5]	
HEG1	[3]	
HELLS	[5]	
HFE	[5]	
HIGD1B	[7]	
HIPK1	[25]	
HJURP	[5]	
HLA-B	[4]	
HLF	[5]	
HLTF	[5] [12]	
HMBS	[4]	
HMGA1	[5] [3]	
HMGA2	[5]	
HMGB2	[5] [12]	
HMGCL	[13]	
HMMR	[5]	
HNF1B	[3]	
HNRNPA 3	[5] [2]	
HNRNPA 3P1	[5] [2]	
HNRNPD	[12]	
HNRNPR	[5]	
HNRPDL	[5]	
HOXA4	[9]	
HOXC8	[14]	
HOXD1	[3]	
HPCAL1	[4]	
HPN	[3]	
HPRT1	[5]	
HRSP12	[25]	
HSD17B1 1	[13]	
HSP90AB 1	[14]	
HSPA1A	[5] [3]	
HSPA1B	[5]	
HSPA8	[4]	
HSPD1	[14]	
HTATSF1	[5]	
HTT	[11]	
HYAL1	[3]	

Gene Cards ID	Ref[#]	no U133A probesets
HYAL2	[3]	
ICAM2	[3]	
ID1	[3]	
ID3	[3]	
IDI1	[3]	
IDUA	[2]	
IFI6	[13]	
IFRD2	[22]	
IGBP1	[5]	
IGF2BP3	[6]	
IGHA1	[13]	
IGHG3	[3]	
IGHM	[3]	
IGKC	[13]	
IGL@	[13]	
IGLJ3	[14] [3]	
IGLL1	[22]	
IGLV6-57	[13]	
IK	[5]	
IKBKB	[11]	
IKZF1	[12]	
IL11RA	[13]	
IL2RB	[25]	
IL6	[9]	
IL6ST	[14]	
IL9	[2]	
ILF3	[5] [17] [2]	
INHA	[5] [22] [12] [4]	
INHBA	[11]	
INPP5B	[5]	
INSIG1	[3]	
INSR	[12] [2]	
INSRR	[11]	
IP6K1	[4]	
IQCG	[7]	
IQGAP1	[14]	
IRF2	[22]	
IRF9	[5]	
IRS1	[12] [11]	

Gene Cards ID	Ref#	no U133A probesets
IRX5	[22]	
ISCU	[12]	
ITCH	[5]	
ITGA2	[4]	
ITGA4	[5]	
ITGA5	[3]	
ITGB3	[12]	
ITGB6	[14]	
ITGBL1	[3]	
ITK	[11] [4]	
ITM2A	[3]	
ITSN1	[12]	
JAG1	[11]	
KAL1	[3]	
KCTD17	[25]	
KDM5C	[3]	
KDR	[11]	
KIAA0020	[4]	
KIAA0101	[5] [2]	
KIAA0317	[4]	
KIAA0754	[5]	
KIF11	[5]	
KIF14	[5]	
KIF15	[5]	
KIF18A	[5]	
KIF18B	[5]	
KIF20A	[5]	
KIF23	[5]	
KIF2A	[5]	
KIF2C	[5]	
KIF4A	[5]	
KIFC1	[5]	
KIR3DL2	[5]	
KLF10	[4]	
KLF6	[5] [4]	
KLF8	[5]	
KLHL7	[5]	
KLRC4	[2]	

Gene Cards ID	Ref#	no U133A probesets
KLRK1	[2]	
KNTC1	[5]	
KPNA2	[5]	
KRAS	[11]	
KRT18	[4]	
KRT19	[4] [1]	
KRT7	[4]	
KRT8	[1]	
KYNU	[4]	
L1CAM	[2]	
LAD1	[3]	
LAMA3	[3]	
LAMA4	[3]	
LAMB1	[4]	
LAMB2	[3]	
LAMP3	[3]	
LANCL1	[2]	
LAPTM4B	[25]	
LARS2	[12]	
LDB2	[3]	
LGALS4	[3] [2]	
LHCGR	[12]	
LIG1	[5]	
LIN28	[25]	
LIN7C	[5]	
LIPE	[5]	
LMAN1	[5]	
LMF1	[5]	
LMNB1	[5]	
LMNB2	[5]	
LMO2	[9]	
LOC100128467	[5]	
LOC100130932	[5]	Yes
LOC146880	[5]	
LOC285359	[5]	Yes
LOC389842	[5]	
LOC399942	[5]	

Gene Cards ID	Ref#	no U133A probesets
LPL	[3]	
LRIG1	[5]	
LRP1B	[11]	
LRRC31	[5]	
LRRC48	[7]	
LRRC50	[7]	
LSM5	[5]	
LST1	[12]	
LTB	[3]	
LTBP3	[5]	
LTBR	[9]	
LTK	[11]	
LY6D	[12]	
LYL1	[2]	
LYPD3	[5]	
MAD2L1	[5]	
MALL	[3]	
MAOA	[5]	
MAP1A	[22]	
MAP2K6	[11]	
MAP3K12	[22]	
MAP4	[3]	
MAP4K1	[12]	
MAP7	[1]	
MAPK1	[2]	
MAPK10	[12]	
MAPK14	[12]	
MAPK8	[11]	
MAPK8IP3	[3]	
MAPKAPK2	[14]	
MARCO	[3]	
MARK2	[2]	
MARS	[5]	
MAST4	[11]	
MAT2A	[14]	
MATN2	[25]	
MATR3	[14]	
MCL1	[14]	
MCM10	[5]	

Gene Cards ID	Ref#	no U133A probesets
MCM2	[5]	
MCM3	[5]	
MCM4	[5]	
MCM5	[5]	
MCM6	[5] [7]	
MCM7	[5] [25]	
MECOM	[11]	
MED1	[12]	
MED13L	[13]	
MEF2C	[12]	
MELK	[5]	
MERTK	[11]	
MET	[14]	
METTL7 A	[3]	
MFNG	[5]	
MGC4049 9	[25]	Yes
MICALL2	[14]	
MKI67	[5]	
MLF1IP	[5]	
MLLT10	[12]	
MLYCD	[5]	
MMD	[5]	
MME	[3]	
MMP1	[14]	
MMP15	[6] [3]	
MMP7	[3]	
MMP9	[3]	
MOGS	[12]	
MOSPD3	[25]	
MPST	[25]	
MRE11A	[5]	
MRPL11	[5]	
MS4A1	[22] [4]	
MSH3	[4]	
MSH6	[5]	
MSLN	[3]	
MST1R	[22] [11] [3]	
MSX2	[2]	
MT2A	[4]	

Gene Cards ID	Ref#	no U133A probesets
MT3	[2]	
MTA1	[3]	
MTHFD2	[5] [2]	
MUC1	[3]	
MUC4	[13] [7]	
MUC5AC	[5]	
MUSK	[11]	
MVD	[14]	
MX2	[6]	
MXI1	[3]	
MYB	[11]	
MYBL2	[5]	
MYH11	[14] [9]	
MYL9	[3]	
MYO1C	[3]	
MYO5C	[5]	
MYST3	[18]	
NACA	[25] [22] [4]	
NAMPT	[14]	
NCAPD2	[5]	
NCAPG	[5]	
NCAPG2	[5]	
NCAPH	[5]	
NCBP1	[9]	
NCF4	[25]	
NDC80	[5]	
NDUFA6	[25]	
NDUFA9	[9]	
NEAT1	[14]	
NEK2	[5]	
NF1	[11]	
NFASC	[14]	
NFATC3	[5] [12]	
NID1	[12]	
NKX2-1	[9] [3]	
NME2	[9] [4]	
NNAT	[25]	
NNT	[12]	
NOMO2	[5]	
NOMO3	[5]	

Gene Cards ID	Ref#	no U133A probesets
NOTCH3	[12]	
NOTCH4	[11]	
NPAL2	[25]	
NPFFR1	[25]	
NQO2	[13]	
NR1H4	[12]	
NR4A3	[14]	
NRAS	[11]	
NT5E	[3]	
NTRK1	[11]	
NTRK2	[11]	
NTRK3	[12] [11]	
NUAK2	[5]	
NUDT1	[5]	
NUP155	[5]	
NUP205	[5]	
NUP210	[5]	
NUP62	[5]	
NUSAP1	[5]	
OBSL1	[13]	
OGT	[2]	
OLFM1	[12]	
OLFML3	[25]	
OPTN	[3]	
ORC1L	[5]	
OTUD4	[14]	
P2RX5	[4]	
P2RY6	[22]	
P5.8	[5]	Yes
P5-04	[5]	Yes
PABPC1	[13]	
PAICS	[2]	
PAK1	[22]	
PAK3	[11]	
PAK7	[11]	
PARK2	[13]	
PARP1	[5]	
PARP2	[5]	
PBK	[5]	
PBXIP1	[4]	

Gene Cards ID	Ref#	no U133A probesets
PCDHA3	[5]	
PCDHGA12	[12]	
PCNA	[5]	
PCOLCE	[25]	
PDAP1	[4]	
PDCD4	[5]	
PDCD6	[25]	
PDCL3	[5]	
PDE4C	[5]	
PDGFRA	[11]	
PDK2	[2]	
PDPN	[3]	
PDX1	[5]	
PDXK	[5]	
PEBP1	[12]	
PECAM1	[3]	
PEX7	[22] [4]	
PFN2	[12]	
PGCP	[5]	
PGK1	[9]	
PGS1	[5] [14]	
PHKB	[5]	
PHTF2	[5]	
PIGC	[12]	
PIGR	[3]	
PIK3C2G	[11]	
PIK3C3	[11]	
PIK3R1	[12]	
PIK3R2	[11]	
PIM2	[11]	
PIN1	[25]	
PIP5K1B	[3]	
PITX1	[5]	
PKD1	[12]	
PKIG	[3]	
PKNOX1	[12]	
PLA2G7	[13]	
PLD3	[9]	
PLEC	[12]	
PLEK	[13]	

Gene Cards ID	Ref#	no U133A probesets
PLGLB1	[4]	
PLGLB2	[25] [4]	
PLK4	[5]	
PLOD2	[9] [2]	
PLUNC	[16]	
PMM1	[25]	
PMP22	[3]	
PNP	[22] [4]	
PNPLA6	[3]	
POLD3	[4]	
PON3	[3]	
POP1	[25]	
POP7	[25]	
POU5F1	[22]	
PPA1	[25]	
PPAP2C	[3]	
PPBP	[12]	
PPFIBP1	[5]	
PPIF	[4]	
PPM1F	[3]	
PPM1G	[5]	
PPOX	[12]	
PPP2CB	[3]	
PPP2R1B	[5]	
PPP2R4	[12]	
PRC1	[5]	
PRDM13	[13]	
PRDM2	[4]	
PRIM1	[5]	
PRKACA	[12]	
PRKACB	[22]	
PRKACG	[5]	
PRKAR1A	[11]	
PRKCE	[2]	
PRKCH	[3]	
PRKDC	[11]	
PRKG1	[14]	
PRMT1	[5] [2]	
PRMT2	[5]	
PRODH	[3]	

Gene Cards ID	Ref#	no U133A probesets
ProSAPiP1	[3]	
PSEN1	[12]	
PSMA6	[13]	
PSMC3IP	[5]	
PSMD7	[2]	
PSME2	[5]	
PSME4	[5]	
PSRC1	[5]	
PSTPIP1	[5]	
PTEN	[11]	
PTGER3	[12]	
PTGIS	[3]	
PTMS	[3]	
PTPN13	[2]	
PTPN9	[22]	
PTPRB	[3]	
PTPRCAP	[22] [4] [3]	
PTPRD	[11]	
PTPRG	[11]	
PTPRM	[3]	
PTRF	[3]	
PTTG1	[5]	
PVALB	[25]	
PVR	[5]	
PYGL	[12]	
QKI	[3]	
R3HDM1	[5]	
RAB11A	[3]	
RAB27B	[2]	
RAB28	[5] [12]	
RAB4A	[5]	
RABEPK	[2]	
RABL4	[25]	
RACGAP1	[5]	
RAD51AP1	[5]	
RAD54L	[5]	
RAD9A	[12]	
RAE1	[12]	

**Gene Cards ID**	**Ref#**	**no U133A probesets**
RALA	[9]	
RALB	[3]	
RAMP2	[3]	
RAMP3	[3]	
RANBP1	[5]	
RANBP9	[11]	
RAP1GAP	[3]	
RARRES2	[7] [3]	
RASL11B	[13]	
RB1	[11]	
RBL1	[5]	
RBL2	[5]	
RBPMS	[5] [12]	
RBX1	[25]	
RCAN1	[14]	
RCL1	[4]	
RECQL	[5]	
RECQL4	[5]	
REG1A	[4]	
RELA	[4]	
RER1	[2]	
RFC2	[5]	
RFC3	[5]	
RFC4	[5]	
RFC5	[5]	
RFTN1	[22] [7] [4] [3]	
RGL3	[5]	
RGS17	[19]	
RGS6	[5]	
RGS7	[9]	
RHOA	[3]	
RHOB	[14]	
RHOBTB2	[5]	
RHOC	[9]	
RHOH	[13]	
RIF1	[5] [12]	
RIPK1	[11]	
RNASE2	[22]	
RNASEH	[5]	

**Gene Cards ID**	**Ref#**	**no U133A probesets**
1		
RNASEH2A	[5]	
RND3	[4]	
RNF125	[14]	
RNF31	[5]	
RNF5	[5]	
RNH1	[3]	
ROBO1	[11]	
RPE65	[4] [2]	
RPL15	[5]	
RPL27A	[22]	
RPL30	[25]	
RPL34	[22]	
RPL39L	[5]	
RPS14	[12]	
RPS26	[4]	
RPS3	[22] [4]	
RPS6KA4	[11]	
RPS6KB1	[4]	
RPSA	[2]	
RRAS	[3]	
RRM1	[5] [9]	
RRM2	[5]	
RRP15	[5]	
RTCD1	[9] [2]	
RUNX3	[23]	
S100A8	[5]	
S100P	[5] [16] [4] [1]	
SAA4	[12]	
SAFB2	[5]	
SAP18	[5]	
SAR1A	[25]	
SBF1	[5]	
SC4MOL	[4] [3]	
SC5DL	[5]	
SCAMP1	[5]	
SCEL	[3]	
SCFV	[13]	
SCG5	[7]	

**Gene Cards ID**	**Ref#**	**no U133A probesets**
SCGB1A1	[9] [7]	
SCGB1D2	[5]	
SCGB2A2	[4]	
SCTR	[3]	
SCYL3	[4]	
SEC31A	[22]	
SEC61A1	[14]	
SELL	[12]	
SELP	[3]	
SEMA3B	[3]	
SEMA3F	[12]	
SENP5	[5]	
SERPINB2	[14]	
SERPINE1	[25] [14] [4]	
SERPING1	[3]	
SFRS2IP	[14]	
SFRS5	[14]	
SFTPA2	[7]	
SFTPB	[7]	
SFTPC	[7]	
SGCE	[3]	
SGSM2	[5]	
SH3BGRL3	[25]	
SH3BP5	[3]	
SH3GL2	[12]	
SH3GLB1	[3]	
SHCBP1	[5]	
SIK1	[5]	
SIKE	[25]	
SIP1	[5]	
SLC12A2	[1]	
SLC12A7	[25]	
SLC15A1	[12]	
SLC16A4	[5]	
SLC17A4	[12]	
SLC1A6	[4]	
SLC20A1	[4]	
SLC22A18	[3]	

Gene Cards ID	Ref#	no U133A probesets
SLC22A18AS	[3]	
SLC23A2	[5]	
SLC25A17	[25]	
SLC2A1	[5] [22] [12] [4]	
SLC35B1	[12]	
SLC38A3	[11]	
SLC4A1	[5]	
SLC4A3	[13]	
SLC5A5	[5]	
SLC6A3	[25]	
SLC7A1	[12]	
SLC7A6	[9]	
SLC7A7	[3]	
SLC9A3	[25]	
SLC9A3R2	[5]	
SLCO2A1	[3]	
SLCO4A1	[5]	
SMAD3	[5]	
SMC1A	[12]	
SMC2	[5]	
SMG1	[11]	
SMURF1	[5]	
SNRPA	[5]	
SNRPA1	[5]	
SNRPB	[5]	
SNRPF	[5]	
SNRPG	[5]	
SNX1	[12]	
SOD2	[14]	
SON	[12]	
SORBS3	[3]	
SORL1	[5]	
SPAG5	[5]	
SPAG9	[5]	
SPANXB1	[4]	
SPARCL1	[9] [3]	
SPC25	[5]	
SPHAR	[5]	

Gene Cards ID	Ref#	no U133A probesets
SPINK1	[3]	
SPINK5	[3]	
SPINT1	[1]	
SPINT2	[1]	
SPOCK2	[22] [3]	
SPP1	[3]	
SPTBN1	[3]	
SRC	[25] [11]	
SREBF2	[25]	
SRPR	[14]	
SSR2	[12]	
ST13	[25]	
ST14	[1]	
ST3GAL6	[5]	
ST6GAL1	[5]	
ST6GALNAC2	[3]	
STARD3	[4]	
STC1	[5] [14] [12] [4]	
STC2	[5]	
STEAP1	[5]	
STK11	[11]	
STMN1	[5]	
STOM	[9]	
STX16	[5]	
STX1A	[22] [4]	
STXBP1	[5]	
SULT1C2	[13]	
SUMO1	[12]	
SUOX	[12]	
SUPT16H	[14]	
SUPT4H1	[12]	
SYCP1	[25]	
TACC3	[5]	
TACSTD2	[1]	
TAF6	[25]	
TAOK1	[14]	
TBL1X	[5]	
TCEA2	[13]	
TCF21	[3]	

Gene Cards ID	Ref#	no U133A probesets
TCP1	[5]	
TDRD3	[5]	
TEF	[25]	
TEK	[9] [3]	
TERT	[25]	
TFAM	[12]	
TFF1	[1]	
TFPI2	[7]	
TFR2	[25]	
TGFBR1	[11]	
TGFBR2	[9] [3]	
TGIF1	[5]	
TH	[5]	
THOC5	[5]	
TIAL1	[12]	
TIE1	[3]	
TIMELESS	[5]	
TIMM22	[5]	
TK1	[5]	
TKT	[3]	
TLE3	[25]	
TLE4	[5]	
TLK1	[12]	
TM4SF4	[7]	
TMC5	[5]	
TMED10	[9]	
TMED9	[12]	
TMEM109	[3]	
TMEM194A	[5]	
TMEM47	[3]	
TMEM48	[5]	
TMEM87A	[5]	
TMF1	[4]	
TMPRSS11E	[5]	
TMPRSS6	[25]	
TMSB4X	[13] [12] [4]	
TNC	[3]	
TNFAIP6	[4]	

Gene Cards ID	Ref#	no U133A probesets
TNFSF9	[2]	
TNK2	[3]	
TOB2	[25]	
TOP1	[14]	
TOP2A	[5] [7] [6]	
TOP3B	[12]	
TOPBP1	[5]	
TP53	[11]	
TP63	[4] [2]	
TPBG	[4]	
TPD52	[13]	
TPM2	[9]	
TPPP	[25]	
TPPP3	[5]	
TPSB2	[9]	
TPX2	[5]	
TRA2A	[12] [4]	
TRIM29	[1]	
TRIM45	[13]	
TRIM8	[5]	
TRIO	[22]	
TRIP13	[25] [5]	
TRO	[13]	
TROAP	[5]	
TSPAN5	[5]	
TST	[25]	
TTC3	[14]	
TTF1	[9] [3]	
TTF2	[5]	
TTK	[5]	
TTLL12	[5]	
TUBA1A	[5] [2]	
TUBA1B	[5]	
TUBA1C	[5]	
TUBA3C	[5]	
TUBA3D	[5]	
TUBA4A	[9] [4]	
TUBB	[5]	
TUBB2A	[5]	
TUBBP1	[5]	

Gene Cards ID	Ref#	no U133A probesets
TXNDC15	[5]	
TYK2	[11]	
TYMS	[5]	
U2AF2	[5]	
UBA1	[2]	
UBC	[4]	
UBD	[3]	
UBE2C	[5]	
UBE2I	[5] [12] [2]	
UBE2S	[5]	
UBE3A	[12]	
UBL3	[5] [3]	
UBL5	[25]	
UBTF	[9]	
UBXN1	[22]	
UFM1	[14]	
UGP2	[12] [4]	
UPK2	[12]	
UQCRC2	[4]	
UQCRFS1	[5]	
USP10	[14]	
USP48	[3]	
UVRAG	[4] [2]	
VAC14	[2]	
VAMP3	[3]	
VAT1	[3]	
VAV1	[11]	
VCAN	[2]	
VDAC2	[4]	
VEGFA	[4]	
VGLL1	[12]	
VLDLR	[4]	
VRK1	[5]	
VSIG4	[3]	
WASF1	[5]	
WDHD1	[5]	
WDR1	[14]	
WDR12	[5]	
WDR60	[5]	
WFDC2	[3]	

Gene Cards ID	Ref#	no U133A probesets
WFS1	[3]	
WHSC1	[5]	
WNT1	[4]	
WNT10B	[4]	
XAGE1A	[14] [7]	
XAGE1B	[24] [21] [14] [7]	Yes
XAGE1C	[14] [7]	Yes
XAGE1D	[14] [7]	Yes
XAGE1E	[14] [7]	Yes
XPC	[5]	
XPNPEP3	[5]	
YEATS2	[5]	
YIPF6	[5]	
YWHAQ	[20]	
ZDHHC11	[25]	
ZFP36L2	[14]	
ZMYND10	[11]	
ZNF154	[12]	
ZNF3	[25]	
ZNF384	[22] [4]	
ZNF410	[12]	
ZNF562	[25]	
ZNF638	[2]	
ZNF665	[25]	
ZNF702	[25]	Yes
ZWINT	[5]	

[#] REFERENCES for Additional_File4

1. Reed CE, Graham A, Hoda RS, Khoor A, Garrett-Mayer E, Wallace MB, Mitas M:**A Simple Two-Gene Prognostic Model for Adenocarcinoma of the Lung.** *J Thorac Cardiovasc Surg* 2008, **135(3)**:627-34.
2. Guo L, Ma Y, Ward R, Castranova V, Shi X, Qian Y: **Constructing Molecular Classifiers for theAccurate Prognosis of Lung Adenocarcinoma.** *Clin Cancer Res* 2006, **12(11)**:3344-54.
3. Hanada S, Maeshima A, Matsuno Y, Ohta T, Ohki M, Yoshida T, Hayashi Y, Yoshizawa Y, Hirohashi S, Sakamoto M: **Expression profile of early lung adenocarcinoma: identification of MRP3 as a molecular marker for early progression.** *J Pathol* 2008, **216(1)**:75-82.
4. David G. Beer, Sharon L.R. Kardia, Chiang-Ching Huang, Thomas J. Giordano, Albert M. Levin, David E. Misek, Lin Lin, Guoan Chen, Tarek G. Gharib, Dafydd G. Thomas, Michelle L. Lizyness, Rork Kuick, Satoru Hayasaka, Jeremy M.G. Taylor, Mark D. Iannettoni, Mark B. Orringer & Samir Hanash: **Gene-expression profiles predict survival of patients with lung adenocarcinoma.** *Nature Medicine* 2002, **8**:814-824.
5. Director's Challenge Consortium for the Molecular Classification of Lung Adenocarcinoma, Kerby Shedden, Jeremy M G Taylor, Steven A Enkemann, Ming-Sound Tsao, Timothy J Yeatman, William L Gerald, Steven Eschrich, Igor Jurisica, Thomas J Giordano, David E Misek, Andrew C Chang, Chang Qi Zhu, Daniel Strumpf, Samir Hanash, Frances A Shepherd, Keyue Ding, Lesley Seymour, Katsuhiko Naoki, Nathan Pennell, Barbara Weir, Roel Verhaak, Christine Ladd-Acosta, Todd Golub, Michael Gruidl, Anupama Sharma, Janos Szoke, Maureen Zakowski, Valerie Rusch, Mark Kris, Agnes Viale, Noriko Motoi, William Travis, Barbara Conley, Venkatraman E Seshan, Matthew Meyerson, Rork Kuick, Kevin K Dobbin, Tracy Lively, James W Jacobson & David G Beer: **Gene expression–based survival prediction in lung adenocarcinoma: a multi-site, blinded validation study.** *Nature Medicine* 2008, **14**:822-827.
6. Kobayashi K, Nishioka M, Kohno T, Nakamoto M, Maeshima A, Aoyagi K, Sasaki H, Takenoshita S, Sugimura H, Yokota J: **Identification of genes whose expression is upregulated in lung adenocarcinoma cells in comparison with type II alveolar cells and bronchiolar epithelial cells in vivo.** *Oncogene* 2004, **23(17)**:3089-96.
7. Nakamura N, Kobayashi K, Nakamoto M, Kohno T, Sasaki H, Matsuno Y, Yokota J: **Identification of tumor markers and differentiation markers for molecular diagnosis of lung adenocarcinoma.** *Oncogene* 2006, **25(30)**:4245-55.
8. Sato M, Tanaka T, Maeno T, Sando Y, Suga T, Maeno Y, Sato H, Nagai R, Kurabayashi M: **Inducible Expression of Endothelial PAS Domain Protein-1 by Hypoxia in Human Lung Adenocarcinoma A549 Cells Role of Src Family Kinases-dependent Pathway.** *Am J Respir Cell Mol Biol* 2002, **26(1)**:127-34.
9. Jiang H, Deng Y, Chen HS, Tao L, Sha Q, Chen J, Tsai CJ, Zhang S: **Joint analysis of two microarray gene-expression data sets to select lung adenocarcinoma marker genes.** *BMC Bioinformatics* 2004, **5**:81.
10. Wang KK, Liu N, Radulovich N, Wigle DA, Johnston MR, Shepherd FA, Minden MD, Tsao MS: **Novel candidate tumor marker genes for lung adenocarcinoma.** *Oncogene* 2002, **21(49)**:7598-604.

11. Ding L, Getz G, Wheeler DA, Mardis ER, McLellan MD, Cibulskis K, Sougnez C, Greulich H, Muzny DM, Morgan MB, Fulton L, Fulton RS, Zhang Q, Wendl MC, Lawrence MS, Larson DE, Chen K, Dooling DJ, Sabo A, Hawes AC, Shen H, Jhangiani SN, Lewis LR, Hall O, Zhu Y, Mathew T, Ren Y, Yao J, Scherer SE, Clerc K, Metcalf GA, Ng B, Milosavljevic A, Gonzalez-Garay ML, Osborne JR, Meyer R, Shi X, Tang Y, Koboldt DC, Lin L, Abbott R, Miner TL, Pohl C, Fewell G, Haipek C, Schmidt H, Dunford-Shore BH, Kraja A, Crosby SD, Sawyer CS, Vickery T, Sander S, Robinson J, Winckler W, Baldwin J, Chirieac LR, Dutt A, Fennell T, Hanna M, Johnson BE, Onofrio RC, Thomas RK, Tonon G, Weir BA, Zhao X, Ziaugra L, Zody MC, Giordano T, Orringer MB, Roth JA, Spitz MR, Wistuba II, Ozenberger B, Good PJ, Chang AC, Beer DG, Watson MA, Ladanyi M, Broderick S, Yoshizawa A, Travis WD, Pao W, Province MA, Weinstock GM, Varmus HE, Gabriel SB, Lander ES, Gibbs RA, Meyerson M, Wilson RK: **Somatic mutations affect key pathways in lung adenocarcinoma.** *Nature* 2008, **455(7216)**:1069-75.
12. Lu Y, Lemon W, Liu PY, Yi Y, Morrison C, Yang P, Sun Z, Szoke J, Gerald WL, Watson M, Govindan R, You M: **A Gene Expression Signature Predicts Survival of Patients with Stage I Non-Small Cell Lung Cancer.** *PLoS Med* 2006, **3(12)**:467.
13. Roepman P, Jassem J, Smit EF, Muley T, Niklinski J, van de Velde T, Witteveen AT, Rzyman W, Floore A, Burgers S, Giaccone G, Meister M, Dienemann H, Skrzypski M, Kozlowski M, Mooi WJ, van Zandwijk N: **An Immune Response Enriched 72-Gene Prognostic Profile for Early-Stage Non^Small-Cell Lung Cancer.** *Clin Cancer Res* 2009, **15(1)**:284-90.
14. Falvella FS, Spinola M, Pignatiello C, Noci S, Conti B, Pastorino U, Carbone A, Dragani TA: **AZGP1 mRNA levels in normal human lung tissue correlate with lung cancer disease status.** *Oncogene* 2008, **27(11)**:1650-6.
15. Kristiansen G, Schlüns K, Yongwei Y, Denkert C, Dietel M, Petersen I: **CD24 is an independent prognostic marker of survival in nonsmall cell lung cancer patients.** *Br J Cancer* 2003, **88(2)**:231-6.
16. Kim B, Lee HJ, Choi HY, Shin Y, Nam S, Seo G, Son DS, Jo J, Kim J, Lee J, Kim J, Kim K, Lee S: **Clinical validity of the lung cancer biomarkers identified by bioinformatics analysis of public expression data.** *Cancer Res* 2007, **67(15)**:7431-8.
17. Guo NL, Wan YW, Tosun K, Lin H, Msiska Z, Flynn DC, Remick SC, Vallyathan V, Dowlati A, Shi X, Castranova V, Beer DG, Qian Y: **Confirmation of gene expression-based prediction of survival in non-small cell lung cancer.** *Clin Cancer Res* 2008, **14(24)**:8213-20.
18. Kuznetsov V, Thomas S, Bonchev D: **Data-driven Networking Reveals 5-Genes Signature for Early Detection of Lung Cancer.** *Proceedings of the International Conference on BioMedical Engineering and Informatics BMEI* 2008, May 27-30, Sanya, Hainan, China, Vol. 1, 413-417.
19. You M, Wang D, Liu P, Vikis H, James M, Lu Y, Wang Y, Wang M, Chen Q, Jia D, Liu Y, Wen W, Yang P, Sun Z, Pinney SM, Zheng W, Shu XO, Long J, Gao YT, Xiang YB, Chow WH, Rothman N, Petersen GM, de Andrade M, Wu Y, Cunningham JM, Wiest JS, Fain PR, Schwartz AG, Girard L, Gazdar A, Gaba C, Rothschild H, Mandal D, Coons T, Lee J, Kupert E, Seminara D, Minna J, Bailey-Wilson JE, Amos CI, Anderson MW: **Fine Mapping of Chromosome 6q23-25 Region in Familial Lung Cancer Families Reveals RGS17 as a Likely Candidate Gene.** *Clin Cancer Res* 2009, **15(8)**:2666-74.
20. Pereira-Faca SR, Kuick R, Puravs E, Zhang Q, Krasnoselsky AL, Phanstiel D, Qiu J, Misek DE, Hinderer R, Tammemagi M, Landi MT, Caporaso N, Pfeiffer R, Edelstein

C, Goodman G, Barnett M, Thornquist M, Brenner D, Hanash SM: **Identification of 14-3-3θ as an Antigen that Induces a Humoral Response in Lung Cancer.** *Cancer Res* 2007, **67(24)**:12000-6.

21. Sato S, Noguchi Y, Ohara N, Uenaka A, Shimono M, Nakagawa K, Koizumi F, Ishida T, Yoshino T, Shiratori Y, Nakayama E: **Identification of XAGE-1 isoforms: predominant expression of XAGE-1b in testis and tumors.** *Cancer Immun* 2007, 7:5.
22. Zhifu Sun, Dennis A. Wigle, Ping Yang: **Identification of XAGE-1 isoforms: predominant expression of XAGE-1b in testis and tumors.** *Journal of Clinical Oncology* 2008, **26(6)**:877-883.
23. Yanagawa N, Tamura G, Oizumi H, Kanauchi N, Endoh M, Sadahiro M, Motoyama T: **Promoter hypermethylation of RASSF1A and RUNX3 genes as an independent prognostic prediction marker in surgically resected non-small cell lung cancers.** *Lung Cancer* 2007, **58(1)**:131-8.
24. Nakagawa K, Noguchi Y, Uenaka A, Sato S, Okumura H, Tanaka M, Shimono M, Ali Eldib AM, Ono T, Ohara N, Yoshino T, Yamashita K, Tsunoda T, Aoe M, Shimizu N, Nakayama E: **XAGE-1Expression in Non-Small Cell Lung Cancer and Antibody Response in Patients.** *Clin Cancer Res* 2005, **11(15)**:5496-503.
25. Aviel-Ronen S, Coe BP, Lau SK, da Cunha Santos G, Zhu CQ, Strumpf D, Jurisica I, Lam WL, Tsao MS: **Genomic markers for malignant progression in pulmonary adenocarcinoma with bronchioloalveolar features.** *Proc Natl Acad Sci U S A* 2008, **105(29)**:10155-60.
